# Novel Bio-Derived Gels from Jin Shofu Starch for the Removal of Corrosion Products from Iron-Based Artworks

**DOI:** 10.1021/acsomega.6c06659

**Published:** 2026-08-26

**Authors:** Andrea Casini, Lucia Bucciacchio, Teresa Guaragnone, Damiano Bandelli, Rodorico Giorgi

**Affiliations:** † CSGI—Center for Colloid and Surface Science, Via della Lastruccia 3, 50019 Sesto Fiorentino, Italy; ‡ Department of Chemistry “Ugo Schiff”, 9300 University of Florence, Via della Lastruccia 3, 50019 Sesto Fiorentino, Italy

## Abstract

Iron-based artworks, often exposed to harsh environmental conditions that accelerate degradation, require conservation methods that balance effective cleaning with environmental impact. Traditional cleaning techniques, which rely on aggressive chemical agents, can damage both the artworks and the surrounding environment, highlighting the critical need for more sustainable alternatives. In response, this study explores a novel approach utilizing a cross-linked gel matrix derived from Jin Shofu starch, chemically and physically modified, and combined with citric acid, a natural chelating and cleaning agent. The cross-linking process is achieved using sodium trimetaphosphate and sodium tripolyphosphate, which are common in the food industry for modifying starch properties. In addition, the freeze–thaw technique exploits the formation and melting of ice crystals to alter the molecular structure of starch, creating new hydrogen bonds and reinforcing the starch matrix’s internal cohesion. This method eliminates the need for additional chemical agents, further advancing the sustainability of the process. The starch-based gel’s biodegradability and compostability ensure that any waste generated during the cleaning process can be disposed of in an environmentally friendly manner, reducing the overall impact of conservation practices.

## Graphical abstract

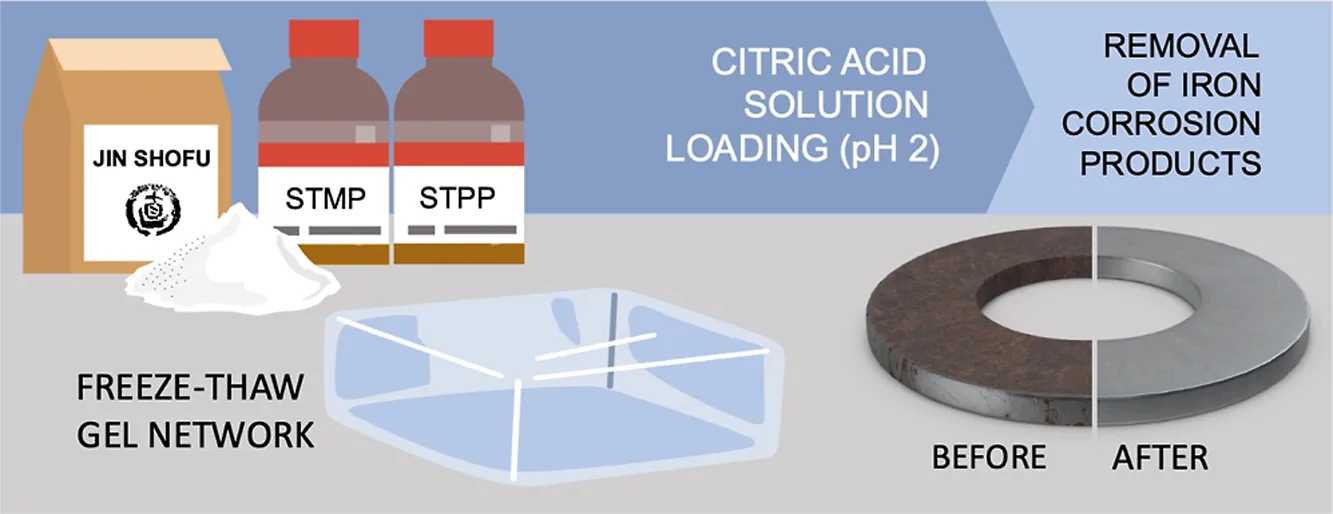

## 1 Introduction

Iron alloys represent valuable materials finding extensive applications in a large variety of industrial and research sectors such as construction, biomedicine, sensors, and more recently in artistic productions.^1^ If, on one hand, the use of ductile iron-based materials can result in thriving artistic experimentation, from grand-scale sculptures to conceptual installations, on the other hand, the constant evolution of contemporary art compels artists and institutions to continuously explore new ways to showcase and share their work with audiences. As a result, artworks made with iron alloys are more frequently exposed to extreme environmental conditions, such as high moisture, pollution, temperature fluctuations, and atmospheric agents that may trigger or accelerate corrosive processes in both indoor and outdoor conditions.^2–4^ Overall, such processes result in the formation of layers of oxidized products, i.e., patinas, that could be historically relevant since they can be connected to the manufacturing technologies and the degradation phenomena experienced but induce significant degradation of these metallic artworks.

Upon corrosion, iron alloys can form several products such as ferrihydrite (hydrated oxyhydroxide of iron), maghemite (γ-Fe_2_O_3_), and α, β, γ-FeOOH (goethite, akageneite, and lepidocrocite).^5^ In particular, the formation of iron hydroxides must be promptly counteracted to avoid the formation of localized pitting that can irreversibly damage the original surface.

Despite the easiness of this concept, the effective restoration of degraded iron-based surfaces represents a complex goal, since degraded layers of iron salts produced by degradation are scarcely soluble.^6^

However, in the last decades, the research of new materials and tools for the conservation of historical artifacts enabled the development of new strategies to counteract metal degradation, resulting in a large variety of materials and techniques.

Among the palette of methodologies employed for the restoration of iron surfaces, the use of gelled systems containing cleaning solutions represents a simple and effective tool to provide fast and controlled cleaning of surfaces. In fact, the synergistic effect produced by the combined use of a target cleaning solution for the solubilization of the corrosion products and a gel, enabling the excellent control of the application area, ensures the development of fast and safe cleaning practices.^7,8^ Similar gel-based strategies have recently been applied to the cleaning of historical and archeological metal artifacts, including bio-organogels for the combined removal of organic coatings and corrosion products from iron heritage^9^ and gel systems for cleaning silver-plated copper alloy archeological coins.^10^ More recently, starch-derived hydrogel systems have been specifically evaluated for artifact-cleaning applications,^11^ further supporting the relevance of starch as a matrix for Cultural Heritage conservation materials. Moreover, gel materials allow for the tuning of the active’s molecular diffusion to maximize the interactions with the degraded layer and to ensure high cleaning performance. Nowadays, an additional, but fundamental feature required for gels and cleaning solution development concerns the overall material’s sustainability and renewability. This requirement addresses the urgent need to align with socio-economic policies such as the European Green Deal promoting the design and the development of eco-friendly materials, preferably obtained via green chemistry approaches to ensure fully sustainable and safe applications.^12^ As a result, the development of fully degradable and renewable gelled systems is an open issue that must be promptly assessed by the combination of sustainable synthetic pathways and tunable material properties such as solvent transport properties, material stiffness, and surficial adaptivity.

In this regard, the fully bio-derived, biodegradable, and sustainable starch represents an ideal candidate to develop advanced materials for Cultural Heritage scopes and aims and has been employed for the preparation of some novel nanoparticles or gels (in association with poly­(vinyl alcohol) (PVA)).^13,14^ For the best of our knowledge, gel systems fully obtained from starch feature poor mechanical properties, limiting their use in Cultural Heritage and in all the technological fields concerning the development of surficial interactions. However, even a simple functionalization of starch molecules could be beneficial to promote the preparation of gels by adding new bonds/functionalities, which include cross-linking. Accordingly, chemical cross-linkers act on the polymeric architecture, promoting the variation of thermal, mechanical, and degradation properties, thus favoring the formation of a three-dimensional network.^15–20^ This trend reflects a broader expansion of starch-based hydrogel research across biomedical, agricultural, and environmental applications. This growth has been recently reviewed.^21^

Herein, we report on the preparation of stable starch gels obtained from the combination of both chemical cross-linking, employing sodium trimetaphosphate (STMP) and sodium tripolyphosphate (STPP) as cross-linking agents,^22^ and physical cross-linking, via freeze–thawing approaches (Scheme 1).

**Scheme 1:**
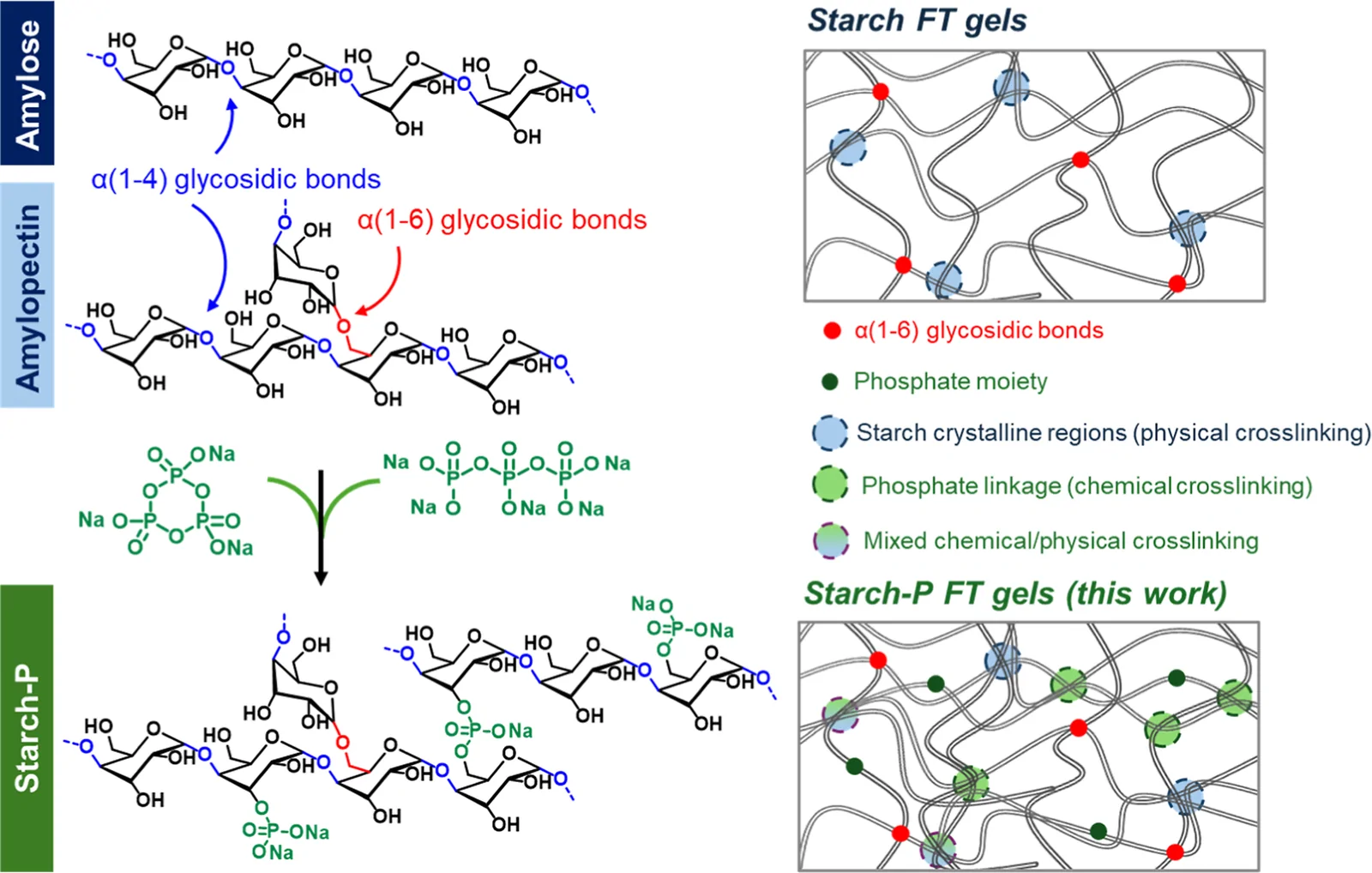
Representation of the Cross-Linking/Modification of Starch and Depiction of the Final Gel’s Structural Features Proposed in the Current Work

Briefly, freeze–thawing approaches are already employed for the preparation of PVA cryogels^23^ and allow the formation of phase separation during the freezing of the free water contained in the mixture. The formation and growth of water crystals induce the formation of crystallites that act as physical cross-link points within the polymer network. As the frozen starch mixture is thawed, the ice crystals melt back into water while polymer crystallites are maintained.

Overall, this elegant but simple process combining both chemical and physical methodologies can be leveraged to develop an innovative hybrid starch-based scaffold that, besides being capable of uploading cleaning solutions, fully complies with sustainable and zero-impact goals.

Aiming to employ the final materials for the removal of iron corrosion products, the gelled systems were charged with citric acid, i.e., a bio-derived, safe ligand for both the complexation and dissolution of iron corrosion products containing oxides/hydroxide species and for its capability of removing dirt/soil from surfaces.^24–26^ Finally, the application on an aged iron alloy was tested and carefully analyzed by means of micro-FTIR and SEM–EDX to evaluate the effectiveness of such materials.

## 2 Materials and Methods

### 2.1 Materials

The Jin Shofu starch used for this study is produced by Paper Nao (Tokyo, Japan). Potassium hydroxide (KOH, ACS reagent, ≥85%, pellets, CAS Number: 1310-58-3), STPP (Na_5_O_10_P_3_, technical grade, 85%, CAS Number: 7758-29-4), STMP (Na_3_O_9_P_3_, ≥95%, CAS Number: 1403834-74-1), and citric acid (C_6_H_8_O_7_, Sigma-Aldrich, CAS Number: 77-92-9) were purchased from Sigma-Aldrich.

Cleaning tests were performed on broad-based washers (10.5 mm × 40 mm galvanized steel, FAI) artificially aged using hydrogen peroxide (H_2_O_2_, ACS reagent, 30 wt % in H_2_O, Sigma-Aldrich, CAS Number: 7722-84-1), sodium chloride (NaCl, ≥99% purity, Sigma-Aldrich, CAS Number: 7647-14-5), and hydrochloric acid (HCl, ACS reagent, 37 wt % in H_2_O, Carlo Erba, CAS Number: 7647-01-0). Water was purified with a Millipore MilliRO-6 Milli-Q gradient system (resistivity >18 MΩ cm).

### 2.2 Starch-Based Hydrogel Preparation

For hydrogel preparation, starch and water are kept at 98 °C for 3 h in a bottom flask equipped with a condenser. Subsequently, the pH was adjusted to 11.5 with an aqueous solution of KOH (2 M), and the polyphosphates were added. The obtained pregel solution was cooled to a temperature of 40 °C under vigorous stirring with a syringe rod. After 1 h, an opalescent, viscous, and homogeneous solution was obtained,^14,27,28^ poured into plexiglass molds (*V* = 14 × 11 × 0.2 cm^3^) and frozen at a temperature of −25 °C. After 24 h, the gel was thawed at room temperature (*T* ≃ 20 °C). Freeze–thaw cycles were repeated under the same conditions, 1 to 3 times (1FT, 2FT, and 3FT). The obtained hydrogels were placed in water to allow the polymeric fraction not stably involved in the newly formed 3-dimensional lattice to exit the structure; all hydrogels were stored in a refrigerator (*T* = 4 °C), and the storage water was changed daily for 3 days.

All syntheses were carried out with STMP and STPP in a ratio of 99:1 (w/w), considering that at alkaline pH (11.5), STMP is a more effective phosphorylating agent than STPP.^29^

### 2.3 Physico-Chemical Characterization of Starch-Based Hybrid Gels

Gel fraction (*G* %) expresses the percentage of polymers stably bound to the three-dimensional lattice of the gel; it was estimated gravimetrically considering the ratio between the weight of the residual dry matter of a gel sample, washed for 72 h, and dried in an oven at a temperature of 100 °C, until constant weight was reached (*W*_d_) and the initial polymer content of the same gel (*W*_0_)­

$$G\;\%=\left(W_{d}{/W}_{0}\right)\times 100$$

*G* % data, as the average of three measurements, are reported in Table 2. Water release (WR) values were calculated to evaluate the retentiveness of the hydrogel, a crucial parameter to consider when setting up a new system for cultural heritage applications.

**Table 1:** Summarizes the Composition of the Aggressive Corrosion Promoter (*R*_sol_) Used in Preparing the Aged Steel Mock-Ups

*R*_sol_ components	H_2_O	NaCl	HCl (1 M)	H_2_O_2_ (1.5 M)
wt %	75.6	1.8	3.8	18.8

**Table 2:** Selected Insights on the Formulation Obtained after 1, 2, or 3 Freeze–Thawing Cycles^a^

	composition			
sample name	starch (w/w %)	STMP + STPP (P) (99:1) (w/w %)	nr. of cycles (*n*)	*G* %
1-S_10_P_0_	10	0	1	96 ± 6
2-S_10_P_0_	10	0	2	93 ± 5
3-S_10_P_0_	10	0	3	100 ± 4
1-S_10_P_0.6_	10	0.6	1	79 ± 1
2-S_10_P_0.6_	10	0.6	2	90 ± 16
3-S_10_P_0.6_	10	0.6	3	69 ± 3
1-S_10_P_6_	10	6	1	77 ± 22
2-S_10_P_6_	10	6	2	98 ± 3
**3-S**_**10**_ **P**_**6**_	**10**	**6**	**3**	73 ± 22
1-S_10_P_12_	10	12	1	51 ± 5
2-S_10_P_12_	10	12	2	57 ± 16
**3-S**_**10**_ **P**_**12**_	**10**	**12**	**3**	30 ± 7
1-S_15_P_0_	15	0	1	71 ± 2
2-S_15_P_0_	15	0	2	73 ± 7
3-S_15_P_0_	15	0	3	75 ± 2
1-S_15_P_0.6_	15	0.6	1	65 ± 2
2-S_15_P_0.6_	15	0.6	2	70 ± 2
3-S_15_P_0.6_	15	0.6	3	78 ± 5
1-S_15_P_6_	15	6	1	63 ± 1
2-S_15_P_6_	15	6	2	63 ± 7
**3-S**_**15**_ **P**_**6**_	**15**	**6**	**3**	67 ± 3
1-S_15_P_12_	15	12	1	64 ± 3
2-S_15_P_12_	15	12	2	78 ± 3

a The systems selected for further investigations are in bold.

The experiments were performed by placing fully swollen gel samples (kept for 72 h in water) on Whatman filter paper in a Petri dish covered with a lid (to prevent evaporation of the fluid); the filter paper was weighed before contact with the gels and 30 min after it. The released water was normalized to per unit area. WR values reported throughout this paper are the averages of three measurements.

Morphological analysis of the gels was conducted by scanning electron microscopy (SEM) analysis performed using a field emission gun scanning electron microscope SIGMA (FEG-SEM, Carl Zeiss Microscopy GmbH, Germany) using an acceleration potential of 2 kV and a working distance of 4.0–4.9 mm. Prior to SEM analysis, freeze-dried gel samples were metallized with gold using an Agar Scientific Auto Sputter Coater. Pore size distributions were built from *n* = 120 individually measured pore radii per sample (pooled from 12 SEM micrographs per gel formulation) using Fiji analysis software.^30^ Prior to measurements, images were converted into 8-bit binary format and then processed using the “distance transform watershed” method; this approach allows individual pores within the gel matrix to be distinguished by disentangling touching pores.^31^ Pores intersecting the image edges were excluded to avoid bias in geometrical data processing.

ATR-FTIR analysis was conducted on dry films using a Thermo Nicolet Nexus 870 spectrometer with an MCT (Mercury Cadmium Tellurium) detector. Spectra of all reagents (bulk starch, Jin Shofu; STMP; STPP) and lyophilized gel systems were recorded over 650–4000 cm^–1^ with a resolution of 2 cm^–1^ and 128 scans per spectrum. This technique was used to assess potential changes in the characteristic starch absorption bands resulting from gel synthesis and its subsequent immersion in an acidic solution. The obtained spectra were deconvolved with IGOR data analysis software to determine the area of the three significant peaks at 1000, 1020, and 1050 cm^–1^. The ratios of these peaks (1020/1000 and 1050/1020) were then calculated to provide insights into changes in the short- and long-range structure of starch induced by the synthesis process and the acid treatment. Proton nuclear magnetic resonance (^1^H NMR) spectra were measured at room temperature on a Bruker AVIII400 UltraShield Plus spectrometer in deuterated DMSO. The residual ^1^H peak of the deuterated solvent was used for chemical shift referencing.

Size exclusion chromatography (SEC) measurements were performed utilizing a system composed of a Shodex ERC-3215α degasser connected with a Waters 1525 binary HPLC pump, a Waters 1500 series heater set at 50 °C, a Wyatt miniDAWN TREOS detector, a Wyatt Viscostar-II detector, a Wyatt OPTILAB T-rEX detector, a Shodex precolumn GPC KD-G 4A, and a Shodex column GPC KD-806 M employing a DMSO/DMF (70/30) mixture as the eluent with a flow rate of 0.70 mL min^–1^. Data evaluation was performed according to standard procedures employing ASTRA software.

### 2.4 Preparation and Characterization of Artificially Aged Steel Samples

To simulate a rust layer, broad-based galvanized steel washers were degraded by an accelerated corrosion treatment based on a rust-inducing solution, labeled as *R*_sol_ (see Table 1). The samples, previously soaked in a 1 M aqueous solution of HCl for about 30 min to remove the passivating ZnO coating, are rinsed with demineralized water and dried with compressed air. Then, using a fine-grit sandpaper (Grit K120, LUX-TOOLS), the washers are treated to remove any residual oxides and increase the reaction surface area standardizing the texture of the substrate. This texture served as a benchmark for evaluating the aggressiveness of the subsequent aging and cleaning processes on the original metallic surface.

To induce corrosion, the prepared washers were briefly immersed in the *R*_sol_, air-dried for 15 min, rinsed with Milli-Q water, and dried using compressed air. The washers were then reimmersed in *R*_Sol_ and air-dried for 48 h. This cycle was repeated two times. Following the final cycle, the washers were stored in air until their use in subsequent cleaning tests.

The rust layer obtained following this procedure was characterized by visible light, micro-FTIR analysis, and SEM–EDX. Macrophotographs were taken in visible light using a Canon EOS 5D Mark II Full Frame DSLR camera equipped with a Sigma 105 mm f/2.8 EX macro lens with lights placed on both sides at 45° so as to minimize shadow formation on the sample’s surface.

FTIR analysis on iron-based mockups was conducted using a Cary 620–670 FTIR microscope (Agilent Technologies). Measurements were performed using either a single-element MCT detector or a Focal Plane Array (FPA) 128 × 128 detector. The latter allows the highest spatial resolution currently available to FTIR microscopes. The spectra were recorded directly on the surface of the samples (corroded steel mock-ups or the Au background) in reflectance mode, with open aperture and a spectral resolution of 8 cm^–1^, acquiring 128 scans for each spectrum. The spectral range was 4000–600 cm^–1^ (Single-element) or 4000–900 cm^–1^ (FPA). The FPA detector was used to perform 2D FTIR imaging. A ‘‘single tile” analysis results in a map of 700 × 700 mm^2^ (128 × 128 pixels), and the spatial resolution of each imaging map is 5.5 mm (i.e., each pixel has dimensions of 5.5 × 5.5 μm^2^). Multiple tiles can be acquired to form mosaics.

Additionally, infrared spectroscopy was also used for identifying the artificially corroded products formed on the mock-ups; ATR-FTIR analyses were carried out, as specified in Section 2.4, on fine powder collected from the steel mock-up surface through mechanical action exerted with a scalpel.

The morphological changes induced by the artificial aging on the metal surface were characterized by SEM analysis of steel mockups (before and after aging) without gold sputtering, under the same conditions described in Section 2.3. Composition variations resulting from aging were analyzed using elemental analysis performed with an EDX system coupled to SEM (Oxford Instruments INCA X ACT microanalyzer).

### 2.5 Cleaning Procedure and Evaluation of Efficacy

The cleaning performance of the systems was evaluated through their application on steel mockups, corroded as specified above. Small pieces of selected gels (2 × 1 × 0.2 cm^3^) were loaded with an aqueous solution of citric acid (0.15 M, pH 2), and after gentle drying to remove excess surface fluid, the gel pieces were applied on the model specimens, with mild pressure to promote adhesion to the metal surface. Three subsequent 30 min applications were made on the surfaces; during the treatment, the hydrogels were covered with Parafilm to minimize fluid evaporation from the retentive matrices.

To assess the cleaning effectiveness and the absence of gel residues on the metal surfaces, visible light images, micro-FTIR analysis, and SEM–EDX analysis were carried out directly on the washers, as described in Section 2.5.

## 3 Results and Discussion

Despite its high availability and excellent green metrics, e.g., renewability, degradability, and processing in safe solvents, starch such as Jin Shofu wheat paste finds limited applications for the development of physical gels due to the resulting gel’s poor mechanical properties. To overcome such limitations, the combination of both physical and chemical cross-linking employing polyphosphates is herein reported. Two polyphosphates, namely, STPP and STMP, were employed both due to their high reactivity for the cross-linking of starch^17,32^ and their low hazard classification according to the European Chemical Agency (ECHA). According to ECHA database, both STPP and STMP are used and approved for the preparation of industrially relevant products such as fertilizers, detergency, and water treatment products, making their use in gel’s development a favorable tool to ensure low environmental impact and toxicity.

### 3.1 Hydrogels Design and Evaluation

Hydrogels were prepared varying starch and polyphosphate concentration, as well as varying the number of freeze–thaw cycles, but keeping the STMP/STPP molar ratio to 99:1. In fact, it has been already demonstrated that this ratio provides optimal control over functionalization and cross-linking via phosphorylation approaches of similar natural polyglycosides.^29,33,34^

The synthetic processes were carried out under alkaline conditions (pH of 11.5), which promoted both starch swelling and gelatinization at lower temperatures compared to traditional gelatinization treatments and enabled faster activation of polyphosphates toward functionalization and cross-linking.

Aiming to develop new, eco-friendly materials with tailored properties, a preliminary screening of synthetic parameters was performed by varying the concentration of starch and the starch/polyphosphate molar ratio. To this aim, liquid/gel phase diagrams of the samples obtained after different freeze–thawing cycles, prepared employing a starch content up to 20% w/w and a polyphosphate content up to 12% w/w were prepared and are reported in Figure 1.

**Figure 1:**
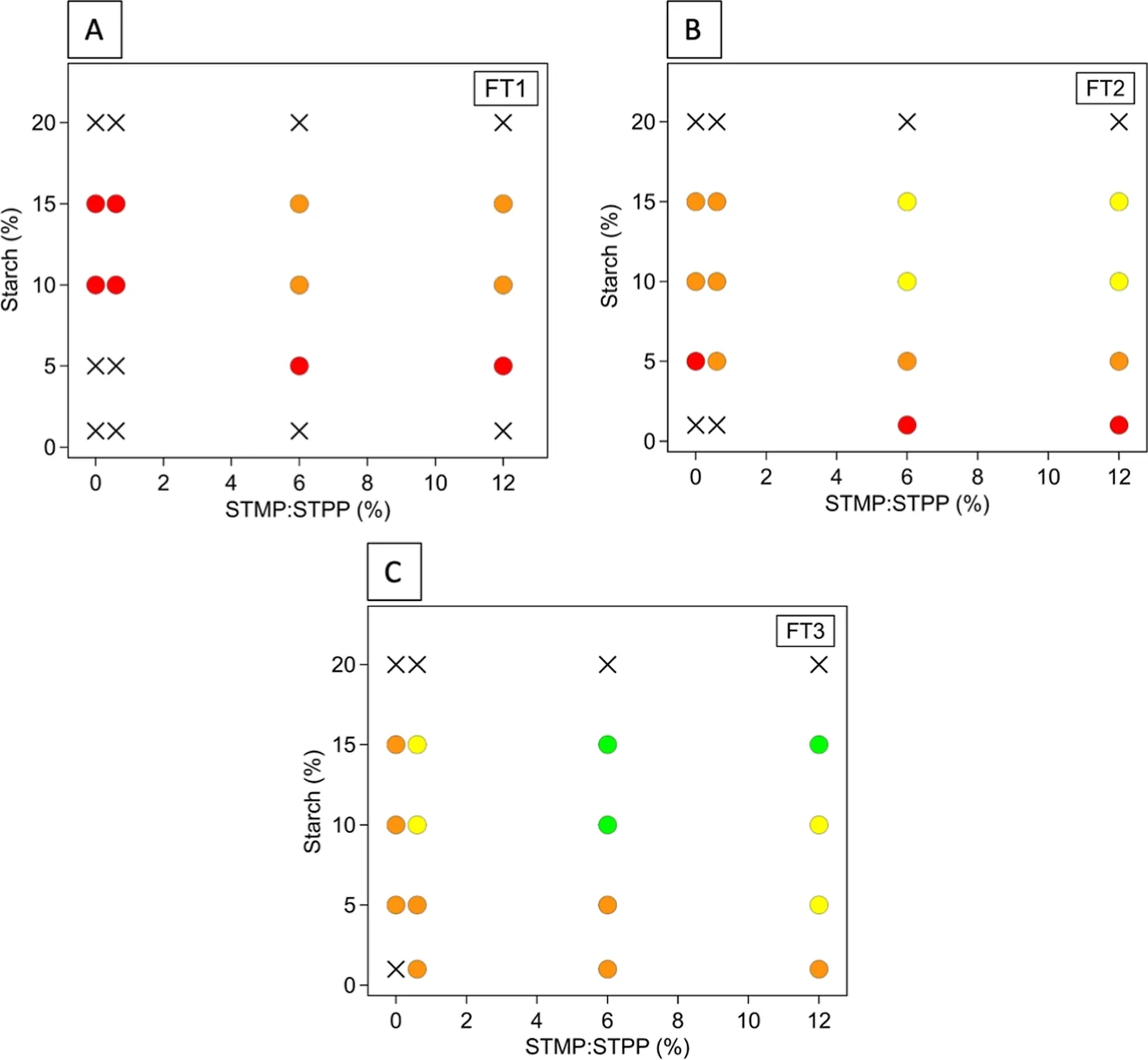
Phase diagrams of the starch formulations after 1 (A), 2 (B), and 3 (C) cycles of freeze–thawing. Crosses represent formulations not employed or not yielding gelled materials, red markers are related to gels with poor mechanical properties, orange-yellow markers are related to average to poor mechanical properties, while green markers correspond to samples with good mechanical properties.

Overall, solutions of Jin Shofu starch at 20% w/w showed high viscosity, making the preparation of the corresponding gels not feasible due to poor handling. Conversely, formulations with a low amount of starch (0 to 5% w/w) resulted in the preparation of poorly stable materials, likely due to low reticulation after FT cycles. In contrast, starch concentrations of 10 and 15% w/w enabled the preparation of physical gels that, upon the addition of polyphosphate cross-linkers and the increment of FT cycles, featured an improvement of mechanical properties.

In fact, an increasing number of FT cycles promotes the formation of a more ordered, denser, and interconnected polymer network, resulting in improved macroscopic characteristics, enhanced elasticity, and mechanical strength.^35–38^

As a result, three systems obtained after three FT cycles and comprising polyphosphates featured gel-like appearances (green dots in Figure 1). Additionally, it is worth mentioning that the gels marked in orange, yellow, and green presented in Figure 1, after being soaked in water to remove any loosely bound polymer fraction, maintain their shape while undergoing swelling.

In Table 2, the names and compositions of starch-based gels are indicated. Each sample was assigned a code of the type *n*-S_*xx*_P_*yy*_, designed to provide a concise indication of its main compositional and synthesis characteristics. Specifically, “*n*” indicates the number of freeze–thaw cycles applied, “*xx*” represents the starch concentration as a percentage of the total system weight, and “*yy*” denotes the polyphosphate content (STMP/STPP 99:1) calculated on the starch dry weight.

Starch-based hydrogels with a gel fraction of 15% w/w exhibit a significant increase in gel percentage (*G* %) with successive freeze–thaw cycles. This behavior can be attributed to the volumetric expansion of water during freezing, which promotes enhanced intermolecular interactions among the starch chains. Consequently, the gel network becomes denser, exhibits reduced swelling capacity, and minimizes the loss of polymer fraction.

In contrast, the wide variability in gel fraction values and the high standard deviation observed in 10% starch-based systems hint toward the presence of heterogeneous polymeric domains within the gel. These domains exhibit varying gel percentage (*G* %) values, depending on the specific region analyzed. The formation of such heterogeneities in 10% starch systems can be attributed to the higher water content compared with that of 15% starch systems.

During synthesis, the cross-linking action of polyphosphates likely targets the starch chains encountered first during dissolution, leading to the formation of highly cross-linked domains. As polyphosphates diffuse further into the system, they interact with regions containing more water and fewer starch chains, resulting in less cross-linked structures that are not uniformly incorporated into the gel’s polymeric network.

When immersed in water, these weakly cross-linked regions swell excessively, compromising the mechanical properties of the system. Consequently, the concentration gradient of cross-linking agents, such as STMP and STPP, during synthesis leads to the coexistence of mechanically robust regions with effective cross-linking and mechanically weaker regions, where cross-linking is not sufficient.

The presence of these heterogeneous domains may raise concerns about the retentivity of the systems. It varies within different regions of the same gel sheet, making them unsuitable for the cleaning of surfaces with historical and artistic importance; in fact, the lack of control over the entire process, coupled with the potential risk of damaging the original art materials, is a significant drawback. To further prove the central role of the polyphosphate cross-linkers in the formation of the starch three-dimensional network, two additional STMP/STPP molar ratios of 50:50 and 1:99 were also tested for the preparation of the 3-S_15_P_6_ gel system. As expected, the final gels obtained by employing an increased amount of STPP cross-linker resulted in poorly stable materials, making their use not appropriate for remedial practices of cultural heritage. This observation confirms that the reactivity of cyclic phosphate is favored due to a release of energy related to the ring opening of the STMP structure during reaction, making cross-linking efficiency more pronounced than its linear counterpart.^39^

Consequently, a system with a starch concentration of 15% was chosen as the best option, given its superior performance and safety compared to those of the 10% starch systems.

### 3.2 Microstructure Characterization

The presence of polyphosphates and their influence on the gels’ microstructure was evaluated through FEG-SEM analysis. Both samples exhibit a honeycomb-like structure characterized by circular and interconnected pores, whose dimensions are related to the presence of polyphosphates. The evaluation of pore size distributions reported in Figure 2 was obtained employing Fiji software as an average of all the SEM pictures comprising more than 5760 pores for each sample.

**Figure 2:**
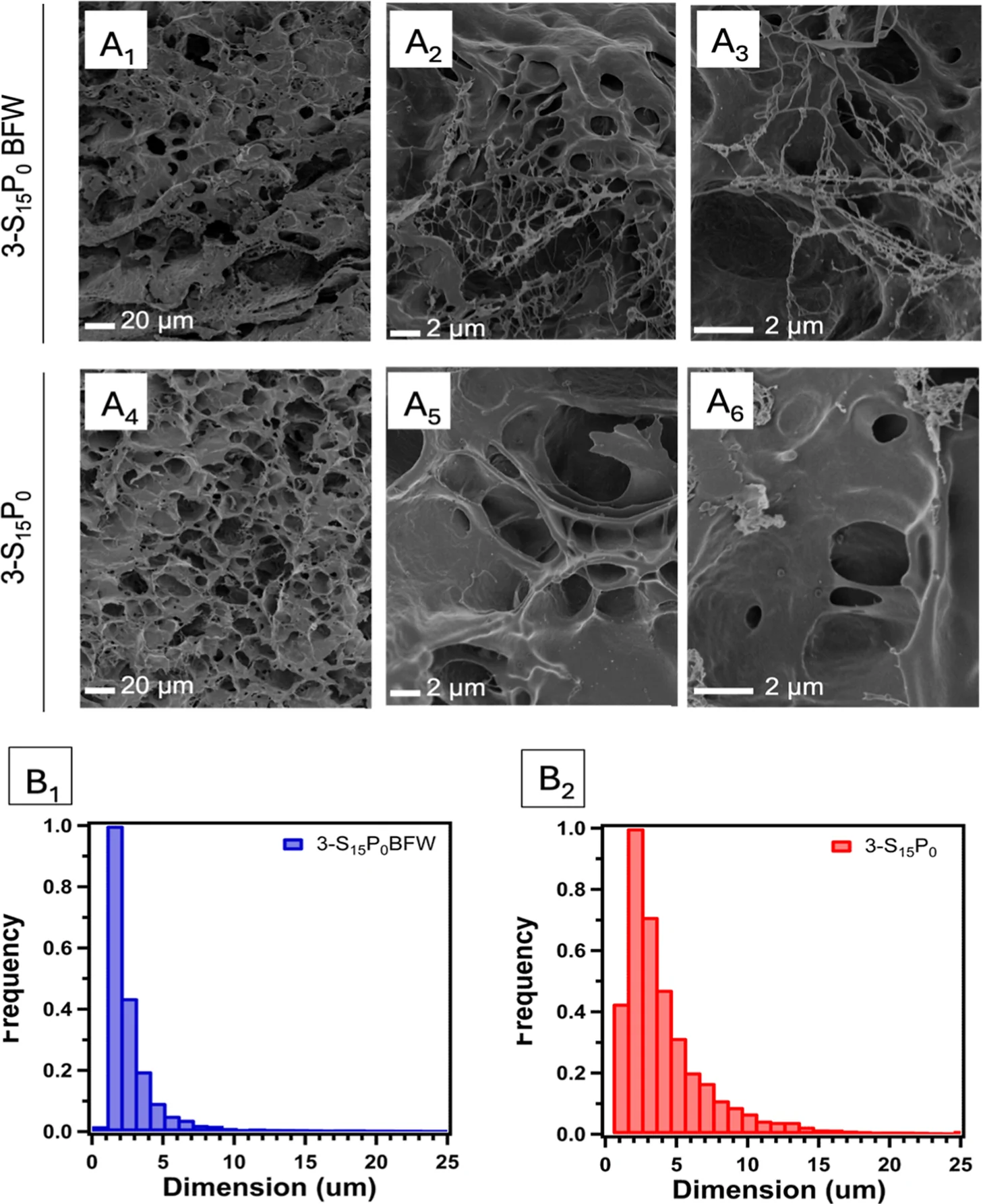
SEM micrographs of the 3-S_15_P_0_ BFW (A_1_–A_3_) and 3-S_15_P_0_ (A_4_–A_6_) systems at different magnifications (600×, 6000×, and 14,000×), along with the corresponding pore size distributions (B_1_ and B_2_, respectively) obtained from image analysis. Each column presents the same magnification for both samples, while magnification increases down the rows. The 3-S_15_P_0_ BFW sample refers to the gel before water washing, whereas 3-S_15_P_0_ corresponds to the same gel after 72 h of Milli-Q water washing.

As a result, the maximum frequency value of the pore size population of the gels decreases from 2.0 to 0.5 μm and features a lower pore size distribution range from 0 to 15 μm for the blank system and from 0 to 10 μm after the addition of polyphosphates.

SEM analysis shows that the addition of polyphosphates leads to a marked reduction in pore size (see Figure 3). The gel walls appear to be closer together, resulting in a more compact and homogeneous structure. This morphological densification correlates with an improved mechanical performance. Rheological measurements reported in the Supporting Information appendix (Figure S1) support these observations, indicating increased rigidity and elasticity of the network. These results suggest that polyphosphates play a structural role within the gel matrix, contributing to the formation of a denser and stronger polymer network.

**Figure 3:**
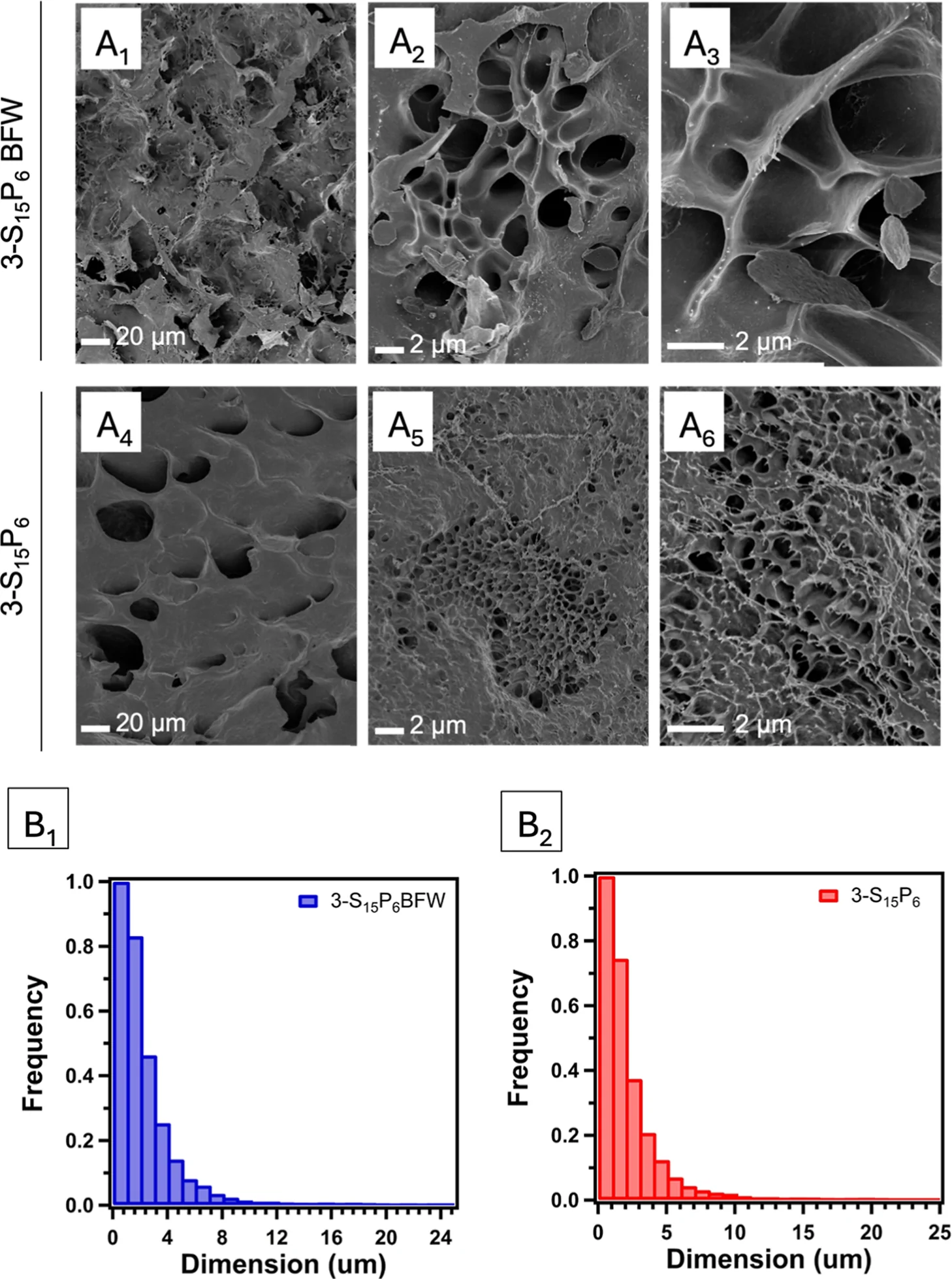
SEM micrographs of the 3-S_15_P_6_ BFW (A_1_–A_3_) and 3-S_15_P_6_ (A_4_–A_6_) systems at different magnifications (600×, 6000×, and 14,000×), along with the corresponding pore size distributions (B_1_ and B_2_, respectively) obtained from image analysis. Each column presents the same magnification for both samples, while magnification increases down the rows. The 3-S_15_P_6_ BFW sample refers to the gel before water washing, whereas 3-S_15_P_6_ corresponds to the same gel after 72 h of Milli-Q water washing.

Additionally, the texture of the 3-S_15_P_6_ system exhibits interconnected pores with well-defined edges, supporting the recorded low WR values and suggesting an improved water-holding capacity and controlled release characteristics.

All gel systems investigated in this study were immersed in water for 72 h. This immersion led to volumetric swelling of the gels, resulting in the expulsion of the nonstably bound polymeric fraction from the newly formed three-dimensional network. Consequently, new pores were formed, or existing pores were enlarged. The development of porosity was verified by comparing the images of washed 3-S_15_P_0_ and 3-S_15_P_6_ gels with those from unwashed samples of the same systems. The SEM images of unwashed (3-S_15_P_0_ and 3-S_15_P_6_) systems revealed the presence of liberated fractions as filamentous structures and isolated gel portions, which were rarely observed in the washed samples.

The effect of the pore size increase can be depicted through the distribution curves representing the diameter-dependent characteristics. In the presence of polyphosphates, the curve did not exhibit a significant shift in its central value. However, in the case of the white gel, a substantial widening of the curve was evident, indicating the formation of pores of diverse sizes. This finding confirms the earlier hypothesis about the role of polyphosphates in governing the morphology of the system. The cross-linking process between chains strengthens the gel structure and generates highly cross-linked polymer domains. During freezing, these domains undergo phase separation tendencies, resulting in textural discontinuities within the system. Subsequently, upon washing, these discontinuities give rise to poorly dispersed porosities with irregular dimensions.

The existing literature supports this hypothesis, demonstrating that gel domains, such as those potentially formed between starch and polyphosphates, can undergo phase separation during freezing. This process influences the templated structure of the systems, which is then disrupted upon being washed. The enhanced porosity and enlarged pore sizes observed in the washed 3-S_15_P_0_ system can be attributed to the release of the weakly bound polymeric fractions, which were not stabilized by the cross-linking process.^14,27^

SEM was also employed to investigate the structural changes induced by loading the cleaning solution in the starch gels (see Figure 4). The resulting distribution curve for the 3-S_15_P_6_ system loaded with citric acid (3-S_15_P_6_ AC) exhibits a slight increase in average pore size, likely stemming from the structural weakening effect of the acidic environment. This led to hydrolysis of exposed starch chains and/or associated polyphosphates, consequently promoting the formation of new pores or the enlargement of existing ones.^40–42^

**Figure 4:**
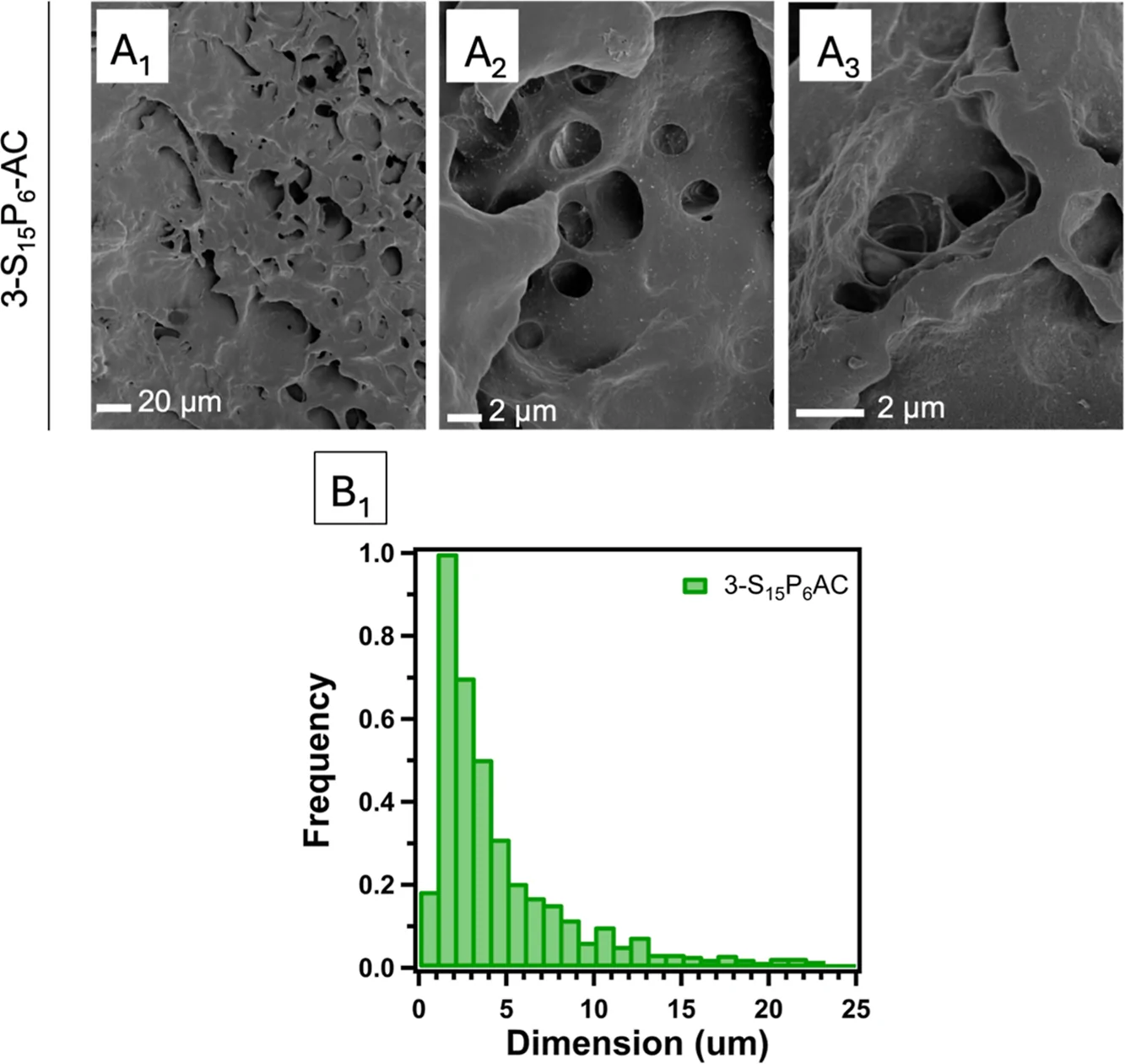
SEM micrographs of the 3-S_15_P_6_ AC (A_1_–A_3_) systems at different magnifications (600×, 6000×, and 14,000×), along with the corresponding pore size distribution (B_1_) obtained from image analysis. The 3-S_15_P_0_ BFW sample refers to the gel after 24 h immersion in citric acid solution.

The edges of the pores in the 3-S_15_P_6_ AC system lacked the sharp and well-defined characteristics compared to the 3-S_15_P gel, indicating a loss of structural integrity. Additionally, the observed changes in pore size and morphology suggest that the gel’s ability to retain and release cleaning solutions may be impacted. Therefore, the immersion time in the acid solution is an important parameter requiring further investigation to optimize the gel’s performance for cleaning applications. However, the available evidence suggests that the use of systems preloaded with citric acid for 24 h does not significantly affect the essential characteristics of the gels required for cleaning applications, such as mechanical properties and retentivity.^40,43^

All gel systems prepared with the addition of STMP and STPP in the reaction environment exhibit distinct chemical–physical, mechanical, and morphological properties compared to those of their respective blanks. This observation suggests that the presence or absence of polyphosphates specifically influences the properties.

### 3.3 The Role of Polyphosphates

The role of polyphosphates in the formulation of the investigated systems was explored by using ATR-FTIR and NMR spectroscopy. ATR-FTIR analysis was conducted on Jin Shofu starch, polyphosphates, and the 3-S_15_P_0_ and 3-S_15_P_6_ systems, with resulting spectra presented in Figure 5, recorded within the spectral range of 650–4000 cm^–1^. In this range, the spectra of Jin Shofu starch and the two analyzed gel systems exhibit characteristic bands associated with the stretching and bending vibrations of the C–H group (located between 2800 and 3000 cm^–1^ and between 700 and 930 cm^–1^, respectively). Moreover, the spectra display bands related to the stretching of the O–H group (3000–3500 cm^–1^), the bending of the C–O–H group (900–1100 cm^–1^, particularly the peak at 995 cm^–1^), and peaks attributed to the stretching of the C–O–H, C–O, and C–C groups (present in the spectral range between 1100 and 1150 cm^–1^).^44,45^

**Figure 5:**
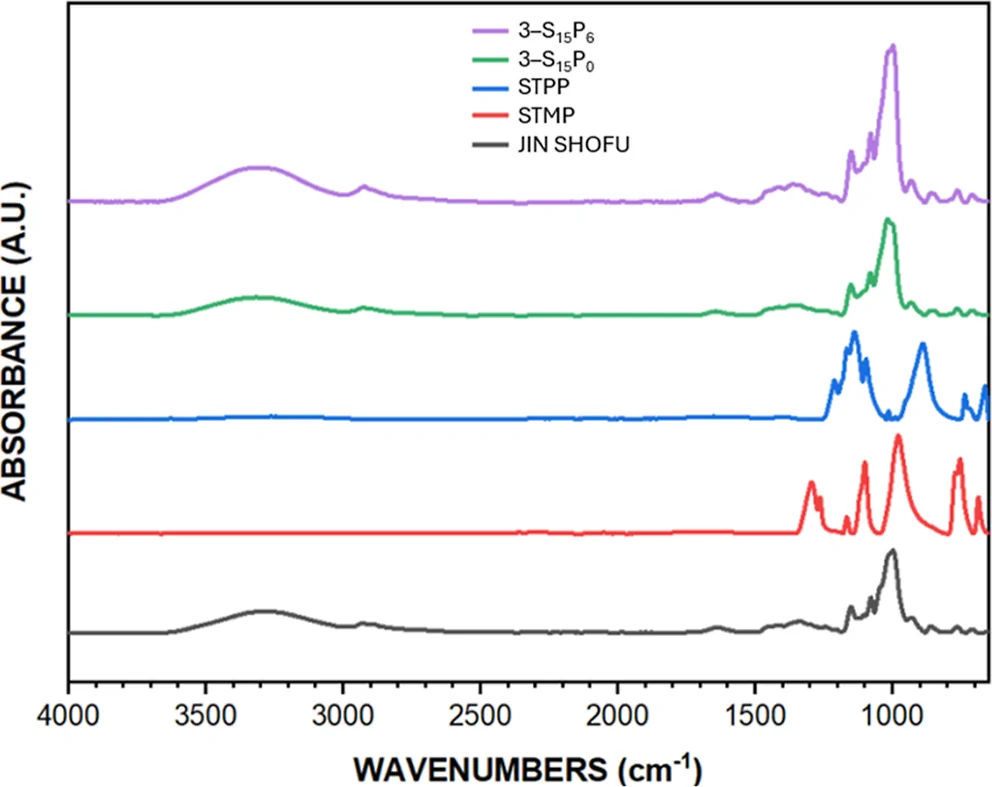
ATR–FTIR spectra of Jin Shofu starch (black), STMP (red), STPP (blue), 3-S_15_P_0_ system (green), and 3-S_15_P_6_ systems (purple).

Interestingly, the ATR-FTIR spectra of the 3-S_15_P_6_ gel system show an absence of the characteristic stretching bands associated with P=O (1100–1220 cm^–1^) and P–O–C groups (around 887 cm^–1^), despite the addition of STMP and STPP to the pregel solution. This suggests that the polyphosphates may act as cross-linkers, reinforcing the gel structure by connecting the starch chains. Furthermore, they are likely responsible for the formation of highly branched or cross-linked polymeric domains. During the freezing process, these domains undergo phase separation within the system, resulting in textural discontinuities that are subsequently lost upon washing, leading to the creation of pores.^27,46,47^

The lack of distinct polyphosphate features in the FTIR spectra of the 3-S_15_P_6_ gel system does not definitively indicate the complete absence of these cross-linking agents within the analyzed samples. To further explore this hypothesis and elucidate the role of polyphosphates in the formation of these gel systems, SEC and proton nuclear magnetic resonance spectroscopy (^1^H NMR) were employed.

At first, SEC analysis of 3-S_15_P_6_ was performed on samples obtained prior to and after swelling in water and was compared to data obtained from the starting Jin Shofu sample.

To this aim, a SEC instrument equipped with multiangle light scattering, viscometer, and refractive index detector was employed to gain insights into absolute molar mass, hydrodynamic radius, and intrinsic viscosity in the mobile phase (DMSO–DMF mixture). The overlay of the chromatograms related to the Jin Shofu employed for formulation, 3-S_15_P_6_BW, the gelled sample before washing in water, and 3-S_15_P_6_ after washing is reported in Figure 6 and suggests an increase of hydrodynamic radius of the samples in the order Jin Shofu <3-S_15_P_6_BW < 3-S_15_P_6_ (Figure 6).

**Figure 6:**
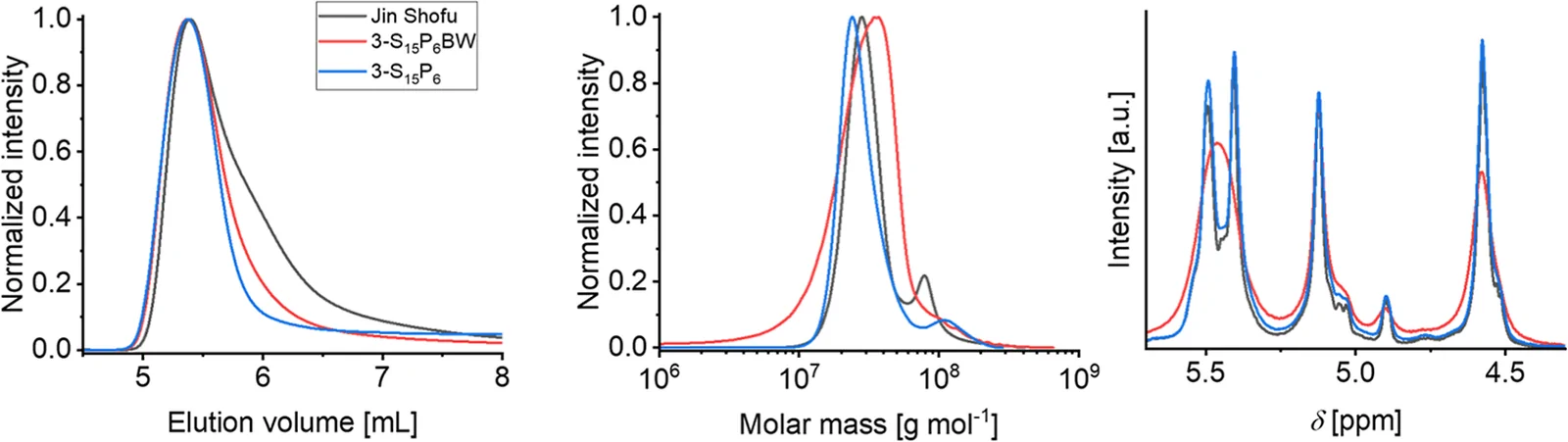
Evaluation of chemical features of 3-S_15_P_6_ and comparison with the initial starch employed for formulation according to SEC and ^1^H NMR analysis. (Left) SEC-MALS chromatograms. (Mid) Molar mass distribution curves from SEC-MALS analysis. (Right) Zoom-in of ^1^H NMR analyses in the region between 4.4 and 5.6 ppm related to the polymer’s signals.

Overall, the SEC analysis revealed an increase of number-average molar mass (*M*_n_) from the starting starch to the polymer gel (3-S_15_P_6_BW), while decreasing the overall molar mass dispersity (D̵) (Table 3). Such outcome suggests that, upon the reaction with the polyphosphate, low molar mass starch was converted to higher molar mass, cross-linked species, resulting in a decrease of D̵. The morphological and architectural variation of starch resulted in the increase of the hydrodynamic radius (*R*_h_) of the final species, while decreasing the intrinsic viscosity ([*h*], i.e., the viscosity of a solution ideally containing one chain of macromolecule). In fact, the increase of molar mass related to interchain cross-linking is followed by the increase of *R*_h_, while the decrease in [*h*] suggests the formation of a densely packed cross-linked species.

**Table 3:** Data from SEC Analysis

	*M*_n_ [10^6^ g mol^–1^]	*M*_w_ [10^6^ g mol^–1^]	D̵	*R*_h_ [nm]	[η] [mL g^–1^]
Jin Shofu	58	170	2.92	64.3	37.935
3-S_15_P_6_BW	62	138	2.22	75.8	31.125
3-S_15_P_6_	58	99	1.69	107.8	58.932

On the other hand, the washing applied resulted in a slight decrease of both *M*_n_ and D̵, suggesting that a polymer fraction has been released from the sample. The evaluation of molar mass distributions reported in Figure 6 suggests that, upon washing, 2 M mass distributions are left in the sample and resemble the initial Jin Shofu composition. To evaluate the nature of such distributions, SEC analyses were performed on certified samples of amylose and amylopectin (see Figure S2 in the Appendix). The analysis clearly indicated that the higher molar mass species were related to the cross-linked amylopectin.

To further investigate the release steps, ^1^H NMR spectra of the three samples investigated via SEC analysis were acquired in DMSO-*d*_6_ at room temperature. Upon functionalization, OH signals at 5.5 ppm collapsed into a broad band, most likely due to hydrogen bonding and polar interactions with phosphate groups (Figure 6, right). Interestingly, the sample collected after the washing step featured a series of signals ascribed to OH groups that can be perfectly superimposed to the initial starch sample.

Overall, the loss of NMR signals related to interactions with phosphate groups, coupled with data obtained from SEC analysis, suggests the loss of cross-linked and phosphate-functionalized species upon washing. Moreover, the slight shift of molar mass ascribed to amylopectin in Figure 6, mid further suggests that, in addition to phosphate-containing polymers, also a fraction of amylopectin has been released.

### 3.4 Long- and Short-Range Interactions in Starch Structural Organization

The cross-linking of starch with polyphosphates is expected to alter the short-range molecular organization of the starch. This is because the phosphate groups can interact with the starch’s hydroxyl groups, disrupting the typical hydrogen bonding patterns.^48^ Additionally, the incorporation of citric acid may also influence the molecular structure, as the carboxylic groups could interact with the starch and polyphosphate moieties, leading to changes in the overall molecular arrangement.^49,50^

Fourier transform infrared spectroscopy is a commonly used technique to analyze the structure of starch, as the IR spectral features in the range of 900 to 1100 cm^–1^ are highly sensitive to variations in the short-range arrangement of starch molecules. Specific bands at 1000, 1020, and 1050 cm^–1^ are commonly referenced in the literature to indicate the presence of amorphous and/or crystalline regions in the analyzed samples. The band at 1020 cm^–1^ exhibits increased intensity in more amorphous samples, while the bands at 1000 and 1050 cm^–1^ become more distinct in samples with higher structural order. Consequently, the ratios of intensities at 1000/1020 cm^–1^ and 1050/1020 cm^–1^ have been adopted as measures of the short-range order of starch molecules. These intensity ratios are higher in amorphous samples and lower in more ordered samples, providing valuable insights into the molecular organization of the starch network.^51,52^

On the basis of the previous consideration of ATR-FTIR data acquired for Jin-Shofu starch, 3-S_15_P_0_, and 3-S_15_P_6_, and considering the presence of a peak related to C–OH stretching at approximately 1720 cm^–1^ in the spectrum of 3-S_15_P_6_ AC, which indicates the presence of residual citric acid as reported in the literature, an attempt was made to assess the changes in structural order induced by the synthesis process, the addition of polyphosphates, and citric acid, in comparison to the initial starch.

The evaluation was conducted by analyzing the ATR-FTIR spectra of Jin-Shofu starch, 3-S_15_P_0_, 3-S_15_P_6_, and 3-S_15_P_6_ AC using data analysis software (IGOR Pro 9.00) to determine the area values of the three prominent peaks at 1000, 1020, and 1050 cm^–1^. The resulting plots from the deconvolution of these spectra are presented below (see Figure 7). The region of interest in the spectrum ranges from 808 to 1184 cm^–1^, and the *y*-axis represents the absorption of the components (shown as dark gray bands), the experimental spectrum (depicted as a black thick line), the fitting curve (represented by a thin white line inside the experimental curve), and the residuals from the fitting (illustrated as a light gray line above the spectrum). The peak at 1000 cm^–1^ is highlighted in green, the peak at 1020 cm^–1^ in red, and the peak at 1050 cm^–1^ in violet. The area values obtained for the significant peaks at 1000, 1020, and 1050 cm^–1^ were utilized to calculate the band ratios at 1000/1020 cm^–1^ and 1050/1020 cm^–1^, which serve as indicators of the short-range order of the starch molecule.^51,52^

**Figure 7:**
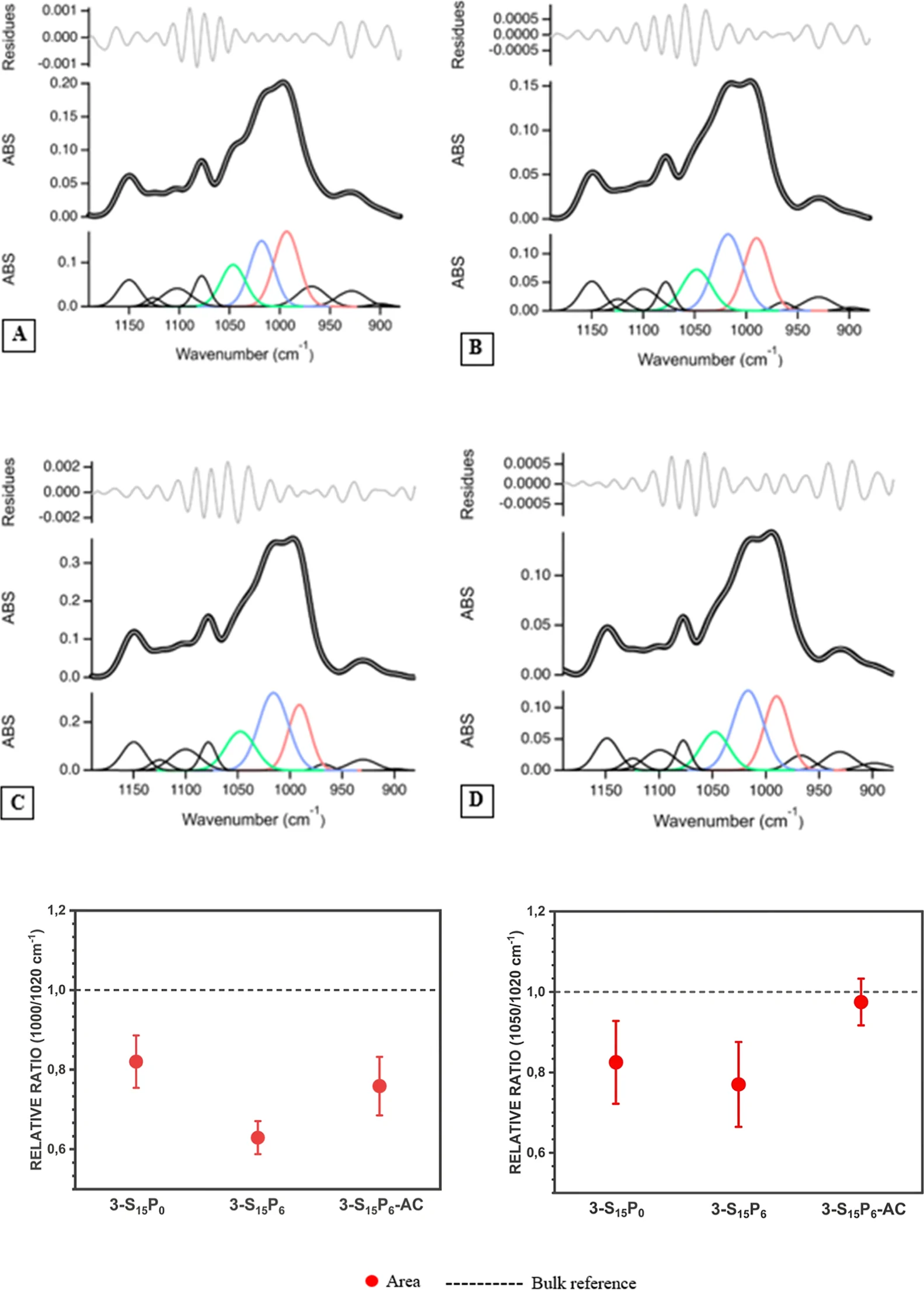
ATR-FTIR spectra deconvolution of starch Jin Shofu (A), 3-S15P0 (B), 3-S15P6 (C), and 3-S15P6-AC (D) and band ratios at 1000/1020 cm^–1^ and at 1050/1020 cm^–1^ of samples 3-S15P0, 3-S15P6, and 3-S15P6-AC.

Normalization of the data obtained for 3-S_15_P_0_, 3-S_15_P_6_, and 3-S_15_P_6_ AC against Jin Shofu starch was performed to facilitate the evaluation of the changes induced by synthesis, polyphosphates, and acid on the structural order of the starting material. The band ratios at 1000/1020 cm^–1^ and 1050/1020 cm^–1^ observed for the 3-S_15_P_0_, 3-S_15_P_6_, and 3-S_15_P_6_ AC systems were found to be lower than those of the reference Jin Shofu starch. This implies that all the investigated systems exhibit reduced short-range structural order compared to the initial starch due to the formulation process and, in the case of 3-S_15_P_6_ AC, the presence of citric acid. Consequently, there is a simultaneous decrease in the long-range order (crystalline order) of the starch, as previously reported in the literature, as the weakening of short-range order structures (double helices) is strongly associated with the disruption of long-range interactions and subsequent destruction of crystallites. Specifically, the observed decrease in the ratios when transitioning from a blank system to one containing STMP and STPP demonstrates the impact of these compounds on the starch structure within the reaction environment. This results in the formation of a system with improved chemical–physical, morphological, and mechanical characteristics, making it more suitable for surface treatments in historical-artistic contexts. The decline in both long- and short-range order can be attributed to the potential presence of polyphosphates acting as “cross-linkers” between starch chains, as well as the formation of cross-linked domains induced by these agents. As mentioned earlier, these domains are released during the washing process, leading to the formation of pores. In both cases, STMP and STPP impede the interaction between starch chains, limiting the formation of double helices and following crystallite growth, thereby resulting in a decrease in the structural order of the system. Conversely, immersion in an aqueous citric acid solution appears to induce the formation of a more highly ordered system. This is evident based on the higher values obtained for the band ratios at 1000/1020 cm^–1^ and 1050/1020 cm^–1^ in the case of the 3-S_15_P_6_ AC system compared to the 3-S_15_P_6_ system immersed exclusively in water. The observed effect can be attributed to the hydrolytic action of the citric acid solution on the starch chains. The hydrolysis leads to the formation of linear segments or segments with weak cross-linking, which possess the ability to interact and form double helices. These double helices, in turn, contribute to the reformation of crystallites within the system. It is well-documented in the scientific literature that the hydrolysis of starch by acids primarily affects the α-(1 → 6) bonds present in the branched structures of amylopectin. This hydrolysis process results in the formation of linear structures that lack branching hindrance, enabling them to readily interact with one another. Consequently, the structural order of the starch is enhanced at both the long-range and short-range scales.^41,53–56^

### 3.5 Cleaning Test

Controlled release and good fluid retention are crucial goals in the synthesis of gel systems, particularly when they are intended for the treatment of surfaces with historical and artistic significance. These properties are fundamental to conservation practice because they mitigate the risks associated with the direct application of cleaning solutions, which can lead to uncontrolled liquid spreading and excessive substrate penetration.^40,43^

The retentiveness of a gel system can be evaluated by quantifying *WR*. However, this measurement can suffer from the uncontrolled detachment of starch pieces from the gel, resulting in an erroneous calculation of WR. Only 3-S_15_P_0_ and 3-S_15_P_6_ gels, having optimum mechanical stability, were analyzed and compared.

Briefly, samples of 3-S_15_P_0_ and 3-S_15_P_6_ were applied for 30 min on Whatman filter paper and exhibited WR values of 5.0 ± 1.0 mg/cm^2^ and 5.8 ± 0.9 mg/cm^2^, respectively.

A comparative analysis of these results with literature values pertaining to PVA-based formulations (11 ± 1 mg/cm^2^), PVA/starch blends (PVA/starch ratios of 2:1, 1:1, and 1:2 with WRs of 14 ± 2, 20 ± 1, and 25 ± 3 mg/cm^2^), pHEMA/PVP gels (15 ± 1 mg/cm^2^), gellan gum, and agar (31 ± 2 mg/cm^2^) indicates that 3-S_15_P_6_ and its “blank” formulation exhibit the highest retentivity among the currently developed systems.^14,27,35^ These results highlight the ability of both systems to maintain a low and controlled solvent release over time, ensuring localized action while minimizing the risks of overwetting or solvent migration. This outstanding characteristic further reinforces their suitability for application on cultural heritage.

Additionally, it is noteworthy that despite only 3-S_15_P_6_ containing STMP and STPP, the recorded WR values for both gels do not significantly differ. This suggests that the incorporation of these components during the synthesis stage does not appreciably affect the final systems’ behavior concerning water or cleaning solution release. However, the increased dispersion of data in 3-S_15_P_0_ reflects a polymer matrix with heterogeneous structures, which locally affect the retentive properties of the gel, thereby contributing to a higher standard deviation value.

The successful removal of corrosion products from a metal surface by using gel systems requires their combination with proper cleaning solutions. In the treatment of iron corrosion products, citric acid was chosen as the complexing agent for iron ions due to its optimal characteristics. These characteristics include low toxicity, minimal hazard to operators, environmental compatibility, cost-effectiveness, ready availability, and efficient performance within relatively short-time applications.

Citric acid is a weak, water-soluble triprotic organic acid (1330 g/L at 20 °C) commonly used in restoration practices, typically as aqueous solutions at concentrations of 5–15%. As a triprotic acid, it undergoes stepwise dissociation, with the third dissociation constant (p*K*_a_3 = 6.4)^57^ being very low, indicating that only a negligible number of molecules undergo ionization.

In solution, citric acid adopts a three-dimensional conformation that allows it to chelate metal ions through its carbonyl and hydroxyl groups of citric acid, allowing it to function as a tridentate ligand. Formation of such coordination complexes requires solubilization of the metal ions, and in the case of rust, this involves the dissolution of iron oxides and hydroxides that have formed because of the corrosion process.^57–59^ These compounds are highly stable at neutral pH (e.g., K_ps_ Fe­(OH)_3_ ≃ 10^–38^),^60^ so their dissolution occurs only in acidic environments. Extensive literature indicates that efficient and rapid solubilization of iron corrosion products takes place at approximately pH 2.^61^ Therefore, the 3-S_15_P_6_ gel was loaded with a 0.15 M aqueous solution of citric acid to facilitate the removal of even the most resistant degradation materials while minimizing the formation of citrate deposits. Similar conditions are commonly employed in commercial rust-removal products and in industrial processes, where dilute HCl solutions are often used to eliminate rust residues and dirt from ferrous materials prior to chromium plating or galvanization. Under these conditions, during the application of the gel, a green-yellow coloration is observed, likely due to the diffusion of iron ions into the gel matrix. This indicates the formation of complexes with citric acid and other salts (e.g., iron chlorides), which are effectively retained by the gel due to its excellent retention properties.

Finally, the 3-S_15_P_6_ system was used to perform cleaning experiments on model specimens prepared according to the procedure described in Section 2. The aim was to evaluate the system’s applicability and effectiveness in removing corrosion products. The cleaning was carried out until the undesired layer was completely removed, followed by rinsing with water to remove any soluble residues (e.g., citrates) and subsequent drying using compressed air. The rinsing step was performed using water-filled 3-S_15_P_6_, providing a controlled application. As shown in Figure 8, macroscopic observations confirmed that the treatment was highly effective, resulting in a uniform metal surface without any residual rust layer.

**Figure 8:**
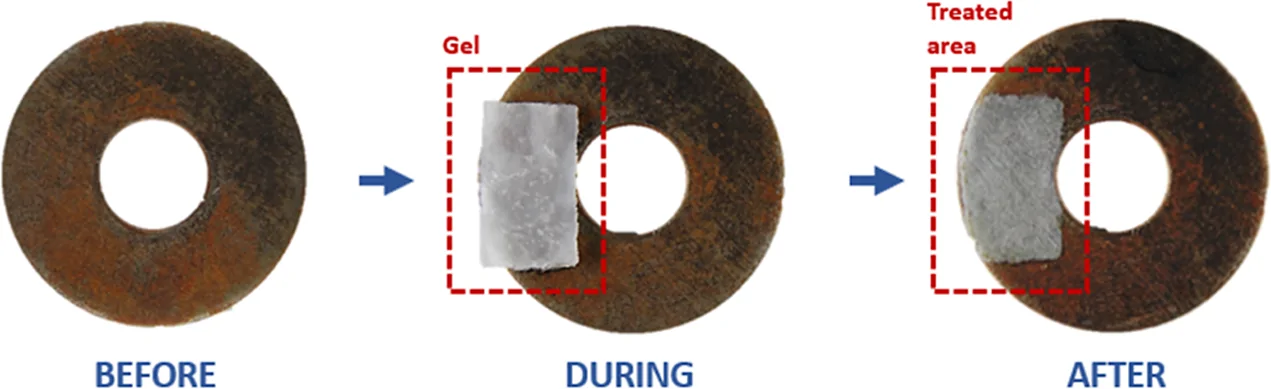
Macro photography of specimens before, during, and after treatments with 3-S_15_P_6_ loaded with citric acid and subsequent rinsing with water.

The effectiveness of the treatment and the presence of any residues were assessed using micro-FTIR and SEM analyses, along with morphological and semiquantitative evaluations. Analysis was performed on the metal surfaces both before the formation of corrosion products and after treatment with 3-S_15_P_6_ loaded with citric acid, allowing a direct comparison of the cleaning efficiency.

2D Fourier-transform infrared (2D-FTIR) measurements were performed within the spectral range of 4000–900 cm^–1^, selecting two specific regions for intensity mapping based on the characteristic absorption bands of corrosion products. The regions 1550–1700 cm^–1^ (Figure 9) and 3300–3500 cm^–1^ (see Figure S3 in the Appendix) were chosen to study OH bending and stretching vibrations, respectively, indicative of hydrated corrosion products like iron hydroxides.^62^ The intensity maps and the corresponding microreflectance spectra of the unaged sample (pristine) confirmed the absence of iron hydroxides across the analyzed surface, and the continuous spectral profile observed is consistent with the metallic nature of the surface, where changes in dipole moment are not readily observable. In untreated, artificially aged samples, micro-FTIR mapping revealed widespread hydroxides across the examined area, with higher concentrations in the red-colored regions. Conversely, the green or blue-colored areas exhibited lower concentrations of hydroxides, likely due to rust products, such as ferrihydrite (Fe_2_O_3_·1/2­(H_2_O)), oxides, or other soluble salts that are readily removed during cleaning. The microreflectance spectrum of the untreated samples displayed a distinct peak around 1650 cm^–1^ corresponding to OH bending, while the 3300–3500 cm^–1^ region associated with OH stretching showed a broad, less defined profile, characteristic of the corrosion product. After cleaning treatment, the micro-FTIR maps and spectrum did not show OH-related peaks or colored regions, indicating that hydrated corrosion products were effectively removed; this confirms the overall effectiveness of the cleaning procedure with the 3-S_15_P_6_ system loaded with citric acid solution.

**Figure 9:**
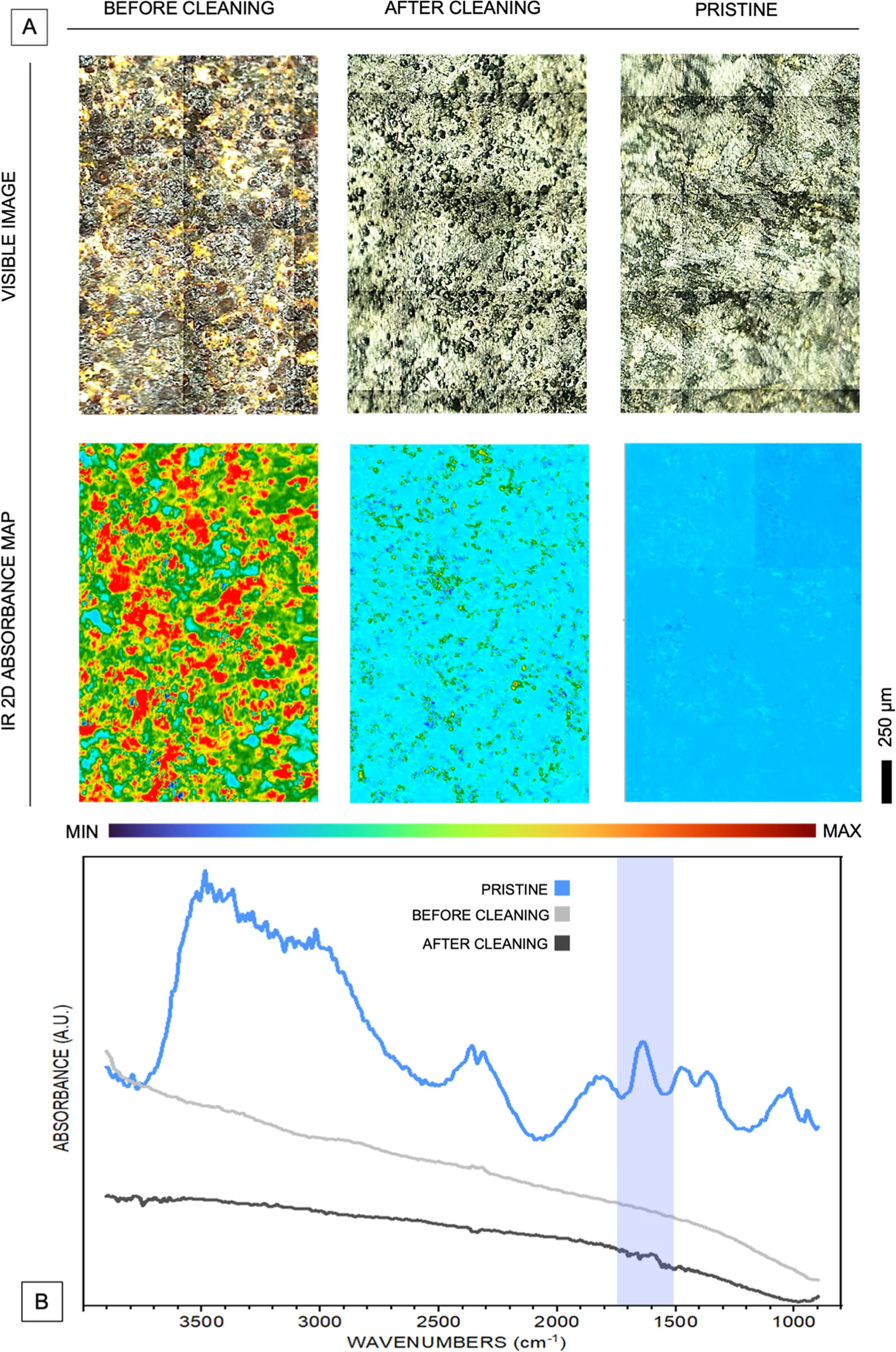
2D-FTIR mapping (A) and FTIR spectra (B) of pristine metal surface compared with artificially corroded sample, before and after cleaning of corrosion products.

Additionally, the 2850–2950 cm^–1^ spectral range, corresponding to C–H stretching, was studied and mapped to assess the possible presence of residues from the gel system (see Figure S4 in the Appendix).^35^ The absence of signals in this region demonstrates that no residual compounds remained on the metal surface after treatment, demonstrating that the 3-S_15_P_6_ system ensures a controlled and effective cleaning action, minimizing the risk of leaving residues that could trigger uncontrolled chemical reactions or promote biological attack.

In the 2D-FTIR intensity maps obtained from both the unaged sample and cleaned sample, localized green—colored areas indicating higher signal intensity compared to the surrounding surface were observed. These regions are likely attributable to interference phenomena rather than the presence of iron hydroxides: the elevated signals appear to correspond to surface cavities, where IR probe scattering may generate locally intensified signals. The same explanation can be given for the variable signal intensities detected in some areas of the untreated and artificially aged samples in the spectral range associated with C–H stretching. This interpretation was supported by SEM–EDX analysis performed on the washer surfaces before aging, as well as before and after cleaning. The analysis of the unaged washer, after removal of the zinc oxide layer, confirmed a low-grade iron–carbon (Fe–C) alloy with 76 ± 5% iron and 23 ± 4% carbon, reflecting the typical composition of inexpensive industrial alloys that are often found in contemporary art. In artificially aged specimens, oxygen and chlorine were also detected. Oxygen is likely due to the formation of iron oxides and hydroxides during the rust formation, while the presence of chlorine is attributed to the use of a corrosion-inducing mixture (*R*_sol_) containing HCl and sodium chloride (NaCl); the concentrations of oxygen and chlorine were determined to be 62.8 ± 0.6% and 0.5 ± 0.2%, respectively. After cleaning, no signal of chlorine or oxygen associated with corrosion was observed, suggesting the complete removal of the corrosion layer without leaving harmful residues. Moreover, phosphorus was not detected on the iron-alloy surface after cleaning, suggesting that phosphate groups are not released from the gel matrix. This is in line with 2D FTIR analysis where no signal related to phosphate groups was detected on the treated surface after cleaning. In other words, the starch-based gels could remove iron oxide patina without releasing their chemical constituents.

Further evidence of the effectiveness of the citric acid-loaded gel system developed in this study for removing corrosion products from iron and its alloys is provided by SEM images. As shown in Figure 10, repeated applications of the gel, following the described protocol, successfully exposed the “original” metal substrate, allowing detailed observation of the chemical—physical and mechanical degradation induced by corrosion on the washer’s surface. The sample displayed a characteristic honeycombing surface morphology, resulting from the autocatalytic corrosive process known as pitting, which produces irregular cavities that facilitate the capillary infiltration of water and chlorides, promoting the initiation and propagation of new pits on the alloy surface. During pitting, metal dissolution creates localized conditions that accelerate the autocatalytic nature of corrosion, allowing pits to spread rapidly throughout the entire metal structure. The severity of pitting is risk associated with pitting corrosion is increased by the accumulation of corrosion products, which exceed the volume of the metal and form a covering at the interface between the anodic and cathodic regions. These coverings can hide underlying damage and make it difficult to assess corrosion macroscopically. At the micrometer scale, these coverings exhibit distinctive morphologies with a central nucleation point from which corrosion products deposit in a radial pattern, with diminishing quantities toward the periphery. The primary aim of this research is the development of an environmentally friendly system for the removal of these corrosion products; consequently, the restored surface retains traces of the degradation process and necessitates protection to prevent further deterioration, which could result in additional damage.

**Figure 10:**
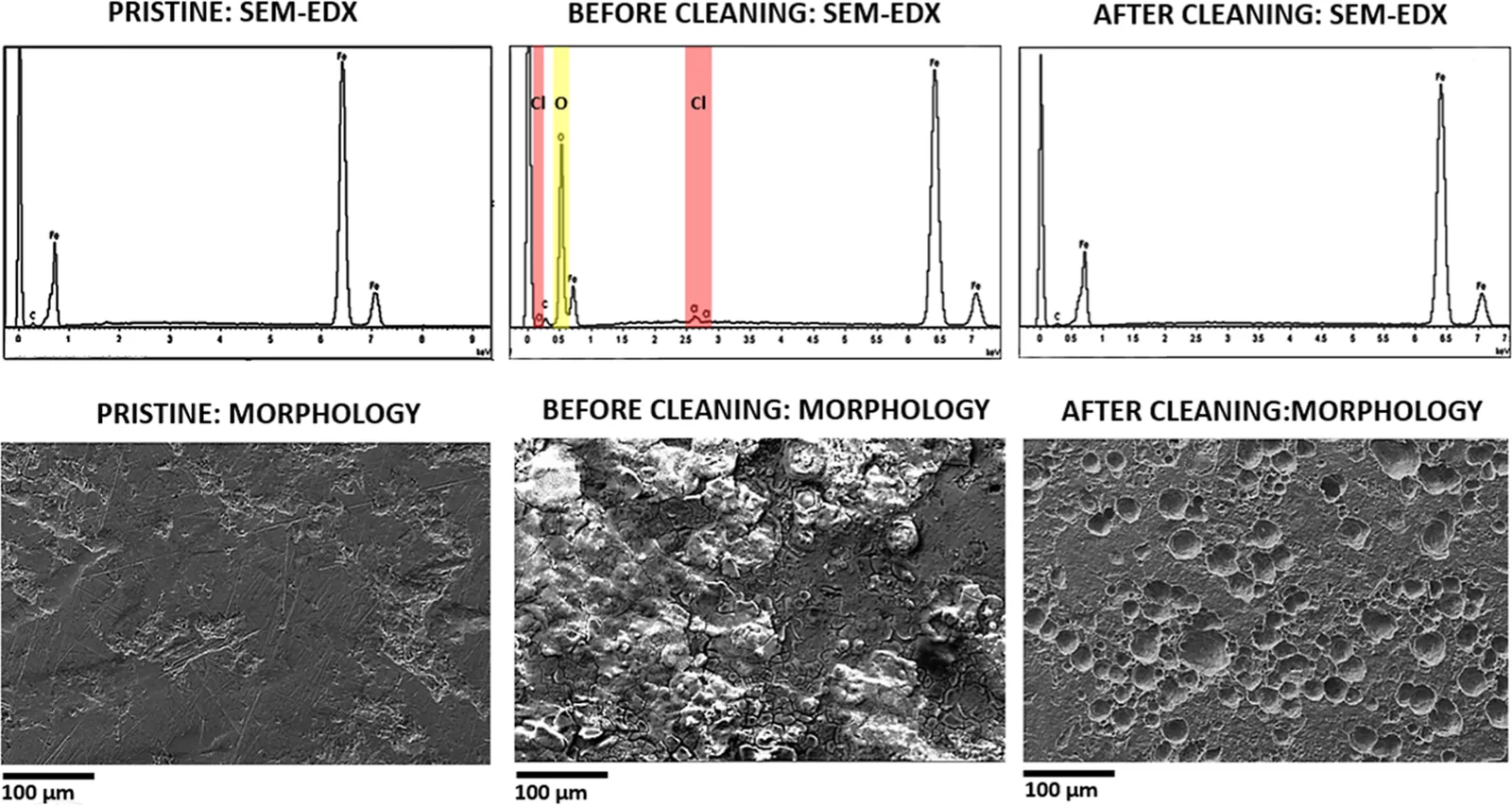
SEM–EDX analysis of pristine metal surface compared with artificially corroded sample, before and after cleaning of corrosion products.

## 4 Conclusions

In conclusion, the use of starch gels loaded with citric acid and cross-linked through a combination of freeze–thaw and chemical cross-linking using polyphosphates offers a green and safe alternative for cleaning iron-based metallic surfaces in contemporary art. Indeed, the gels herein proposed are obtained employing safer chemicals and solvents, raw renewable feedstocks, and, given the degradability of the initial reactants, they are expected to show optimum degradability and compostability.^63^

The gel formulation exclusively incorporates starch and polyphosphates that are commonly used in the food industry, opening for possible application in this industrially relevant sector. Additionally, the gel system owns desirable characteristics such as biodegradability, nontoxicity, and environmental friendliness and sustainability, aligning with the principles of a circular economy. Moreover, starch-based hydrogel matrices of this kind have been proposed as slow and controlled release vehicles for agricultural fertilizers,^64^ suggesting that, after application on iron-based substrates, the citrate/iron-loaded gel residues could represent a candidate feedstock for iron-deficient soil amendment.^65^ This potential application has not been experimentally verified in the present study and is proposed as a direction for future work.

Three distinct procedures were considered to synthesize starch-based gel systems, varying instrumental setups and reaction parameters like pH, temperature, and time. These factors significantly influence the physicochemical and mechanical properties of the resulting gels.

The synthesized gels were evaluated macroscopically, considering factors such as heterogeneity, viscosity, shape retention, and ease of handling. Among the tested systems, the 3-S_15_P_6_ gel was the best candidate in terms of its mechanical stability, gel fraction, and handling. Moreover, it showed high solvent retentiveness, i.e., low WR features, surpassing already established materials for applications on water-sensitive substrates.

Further insights were gained through preliminary investigations on the gels’ behavior in response to variations in reactant concentration and the number of “freeze–thaw” cycles. Increasing the “freeze–thaw” cycles enhanced the mechanical properties by promoting a more structured and interconnected gel network. While higher starch content improved system manipulability, a similar trend was observed for polyphosphates only up to 6% of the dry starch weight; beyond this threshold, increased STPP and STMP led to decreased manipulability.

In all of the synthesis procedures, STMP and STPP were added in a 99:1 ratio. ATR-FTIR and NMR spectroscopy investigations suggest that polyphosphates likely cross-link starch chains, reinforcing the gel structure and creating highly branched and cross-linked polymeric domains. During freezing, these domains tend to undergo phase separation, resulting in textural discontinuities and poorly dispersed porosity observed in the SEM analysis of specific gel samples.

The application of the 3-S_15_P_6_ starch-based gel loaded with citric acid on an artificially aged rust layer demonstrated promising results for the potential use of this system in cleaning iron-based substrates of historical and artistic significance. The treatment effectively removed saline concretions and corrosion products without release of the gel’s constituents, as confirmed through various analytical techniques. The 3-S_15_P_6_ gel system loaded with citric acid exhibited favorable characteristics for conservation purposes, enabling the controlled removal of corrosion without causing chemical or physical damage to the original art materials.

The synthesized starch-based gel, characterized by biodegradability, nontoxicity, and environmental compatibility, shows promising potential for treating a wider range of materials beyond iron-based surfaces. As a biosustainable matrix for the delivery of cleaning fluids, this system could be extended to other cultural heritage substrates. A key next step will be evaluating the feasibility of these gel systems at basic or neutral pH in order to broaden their applicability across diverse artistic and historical materials. Given its adaptable formulation, the gel holds promise as an effective and sustainable tool for the controlled delivery of cleaning fluids to a wide range of cultural heritage objects and artworks.

## Supplementary Material



## Notes

Rheological characterization of the 3-S_15_P_0_ and 3-S_15_P_6_ systems; SEC analyses of certified samples of amylose and amylopectin; micro-FTIR analysis in the 2850–2950 cm^–1^ and 3300–3500 cm^–1^ spectral regions of the treated surface (PDF)
