# Supplementary material for: Novel Bio-Derived Gels from Jin Shofu Starch for the Removal of Corrosion Products from Iron-Based Artworks
Source: ACS Omega. 2026 Aug 26;11(35):53387–403. doi: 10.1021/acsomega.6c06659 (PMC13564001; doi:10.1021/acsomega.6c06659)
Supplement: Supplementary file 1 [file ao6c06659_si_001.pdf]

SUPPORTING INFORMATION

NOVEL BIO-DERIVED GELS FROM JIN SHOFU STARCH FOR THE REMOVAL OF CORROSION PRODUCTS FROM IRON-BASED ARTWORKS

*Andrea Casini[1], Lucia Bucciacchio[2], Teresa Guaragnone[1], Damiano Bandelli[1,2], Rodorico Giorgi[1,2 *]*

[1] CSGI – Center for Colloid and Surface Science, Via della Lastruccia 3, 50019 Sesto Fiorentino, Italy

[2] University of Florence, Department of Chemistry "Ugo Schiff", Via della Lastruccia 3, 50019 Sesto Fiorentino, Italy

***Corresponding author:** rodorico.giorgi@unifi.it

1. Mechanical and rheological characterization

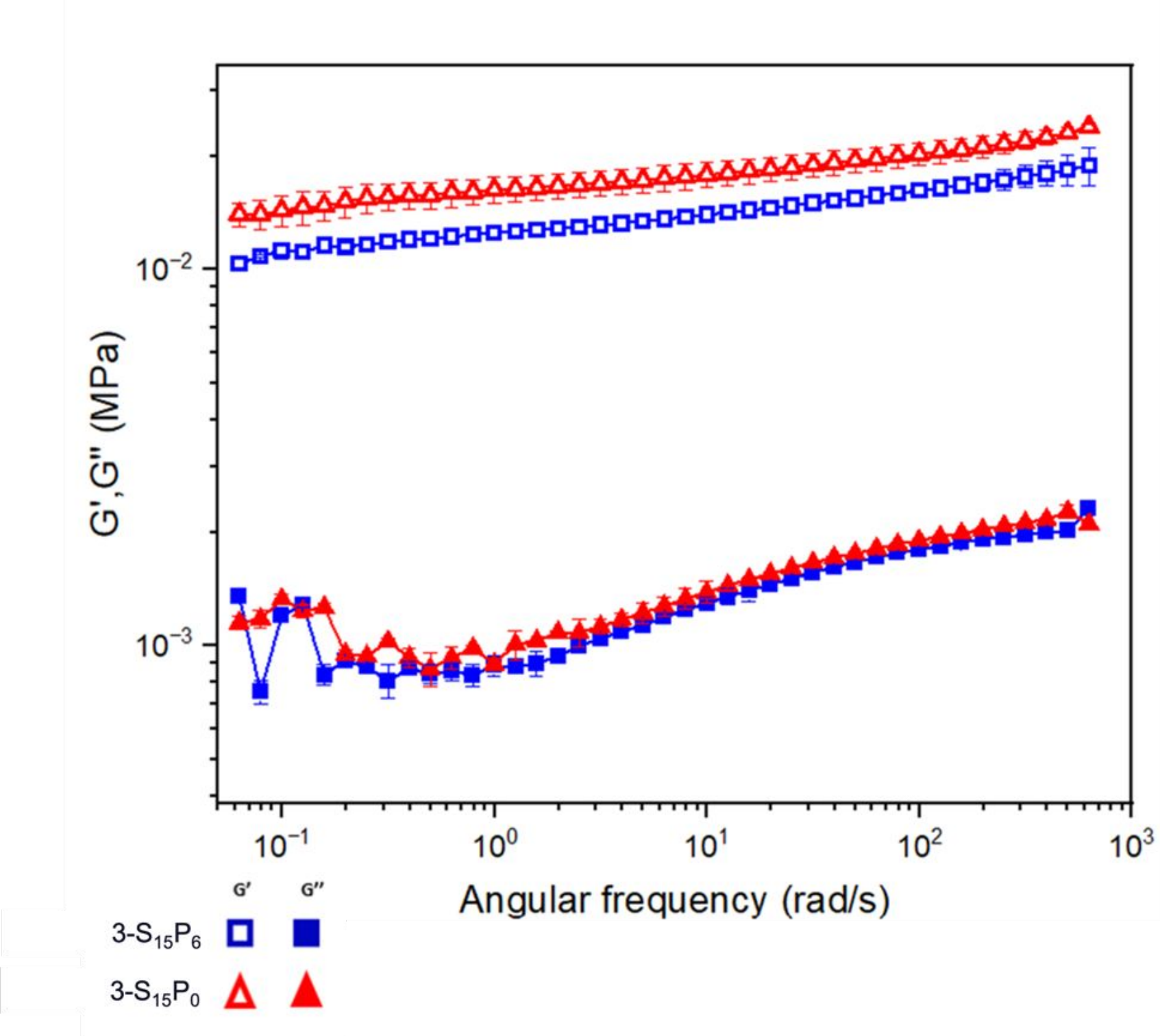

Figura S1. Storage modulus (G') and Loss modulus (G") obtained from frequency sweep tests conducted within the linear viscoelastic region (LVE) of the 3-$S_{15}P_0$ and 3-$S_{15}P_6$ systems.

The mechanical and rheological properties of the 3-$S_{15}P_0$ and 3-$S_{15}P_6$ gels were assessed by comparing the values of G' (storage modulus) and G" (loss modulus) obtained from frequency sweep tests conducted within the linear viscoelastic region (LVE), where the applied force is linearly related to the material deformation. The amplitude sweeps tests indicated that the applied deformation ranged from 0.01% to 0.1%, with a specific value of approximately 0.7%. The increase in G' and G" values observed when transitioning from the 3-$S_{15}P_0$ system to the system with the addition of sodium trimetaphosphate (STMP) and sodium tri-polyphosphate (STPP) during synthesis suggests that polyphosphates play a significant role in determining the mechanical properties of the final gel system. Considering the observations from scanning electron microscopy (SEM), attenuated total reflectance Fourier transform infrared

spectroscopy (ATR-FTIR), size exclusion chromatography (SEC) and nuclear magnetic resonance (NMR) analysis, it can be hypothesized that these differences are attributed, at least in part, to the presence of phosphate bridges between starch chains, even at very low concentrations. These bridges enhance the mechanical resistance of the gel system, enabling it to withstand higher levels of stress.

Furthermore, the distinct morphologies exhibited by these gel systems also contribute to the observed variations. The presence of polyphosphates allows for the formation of highly "cross-linked" domains, which are then lost during the washing process, resulting in the formation of pores. This phenomenon reduces the dispersion of pore sizes and imparts a higher degree of homogeneity to the 3-$S_{15}P_6$ gel system. As a result, this gel system is more able to distribute applied tension and compression stresses evenly across its structure. In contrast, the 3-$S_{15}P_0$ system exhibits dispersed porosity, with larger pores acting as weak points that limit the gel's ability to uniformly absorb and distribute such stresses, leading to decreased mechanical strength.

2. Size exclusion chromatography of amylose and amylopectin reference standards

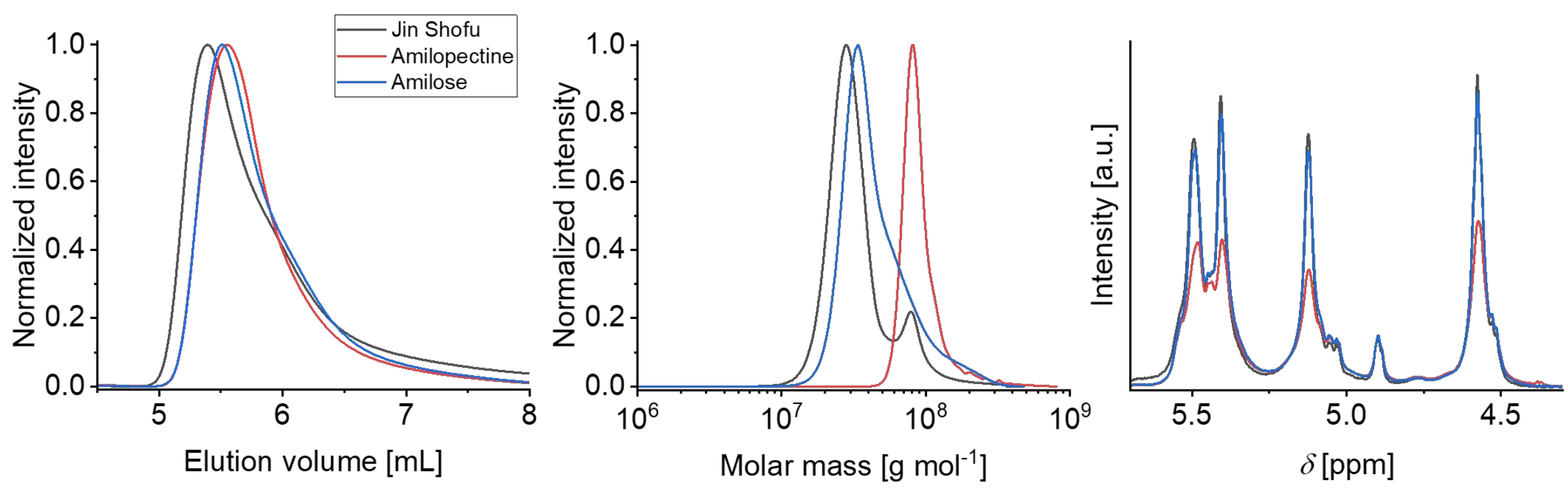

Figure S2. Analysis of Jin Shofu starch (black line), amilopectine (red line) and amylose (blue line). **Left.** Overlay of SEC traces of the three samples employing MALS detection. **Mid.** Overlay of molar mass distributions from the three samples obtained from SEC analysis employing MALS-RI detection. **Right**. Overlay of 1H NMR traces of the three samples in the region between 4.3 to 5.7 ppm (RT, DMSO, 400 MHz).

3. Spectroscopic characterization of the gel before and after application on corroded steel mock-ups

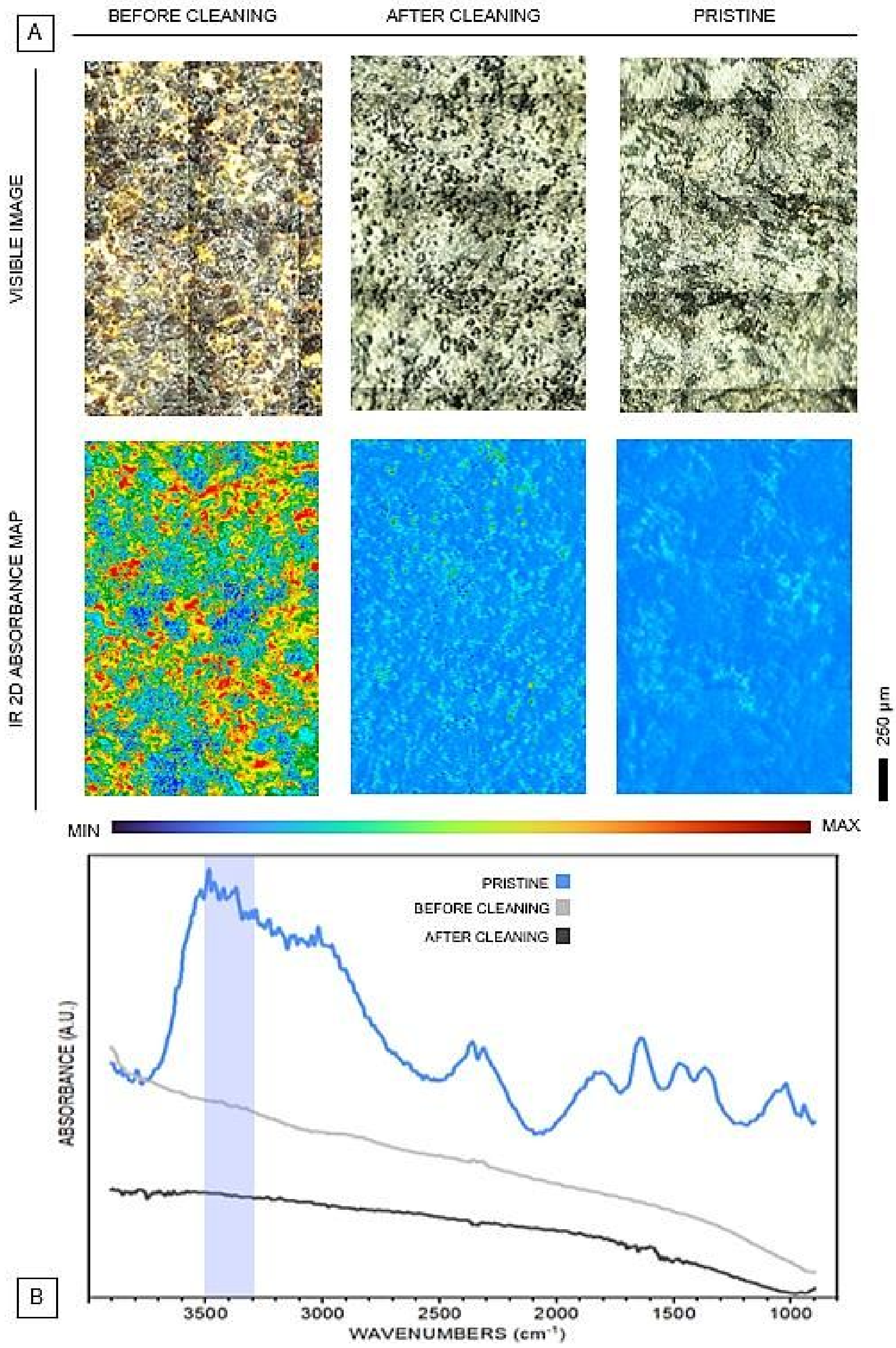

Figure S3. 2D-FTIR absorbance maps (A) and corresponding micro-reflectance spectra (B) acquired in the 3300–3500 cm^{-1} spectral region associated with OH stretching vibrations of hydrated iron corrosion products, for pristine, artificially corroded (before cleaning), and cleaned metal surfaces. The maps confirm the widespread presence of iron hydroxides on the uncleaned surface and their complete removal following treatment with the 3-$S_{15}P_6$ gel loaded with citric acid.

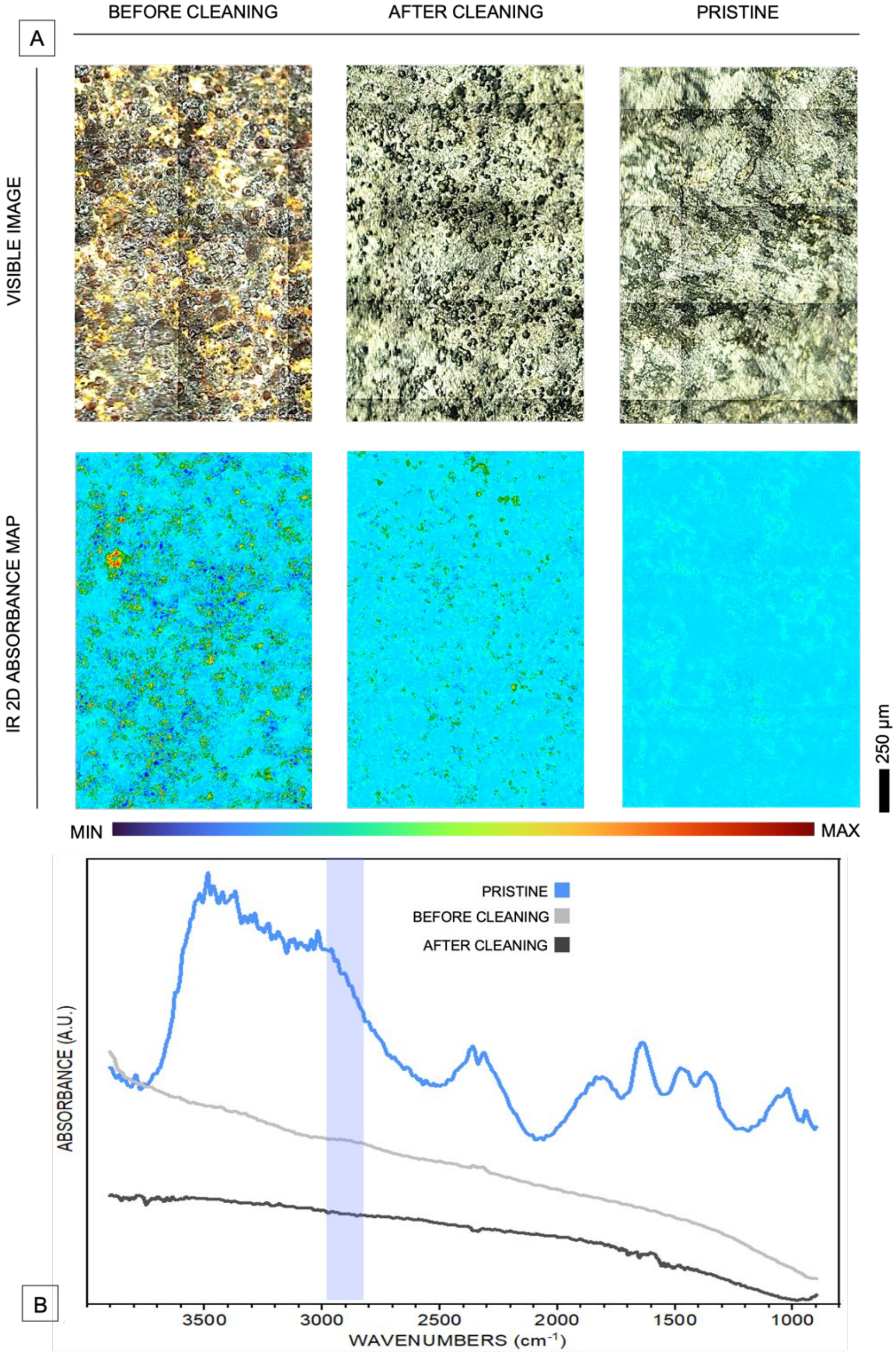

Figure S4. 2D-FTIR absorbance maps (A) and corresponding micro-reflectance spectra (B) acquired in the 2850–2950 cm^{-1} spectral region associated with C–H stretching vibrations, for pristine, artificially corroded (before cleaning), and cleaned metal surfaces. The absence of detectable signal intensity in this region after treatment confirms that no organic residues from

the 3-$S_{15}P_6$ gel system remained on the metal surface following the cleaning procedure and subsequent rinsing step.
